# Deduction learning for precise noninvasive measurements of blood glucose with a dozen rounds of data for model training

**DOI:** 10.1038/s41598-022-10360-3

**Published:** 2022-04-20

**Authors:** Wei-Ru Lu, Wen-Tse Yang, Justin Chu, Tung-Han Hsieh, Fu-Liang Yang

**Affiliations:** 1 Research Center for Applied Sciences, Academia Sinica, 128 Academia Rd., Sec. 2, Nankang, Taipei City, 115-29 Taiwan; 2 Department of Biomechatronics Engineering, National Taiwan University, No. 1, Sec. 4, Roosevelt Rd., Taipei City, 10607 Taiwan

**Keywords:** Diabetes, Information technology

## Abstract

Personalized modeling has long been anticipated to approach precise noninvasive blood glucose measurements, but challenged by limited data for training personal model and its unavoidable outlier predictions. To overcome these long-standing problems, we largely enhanced the training efficiency with the limited personal data by an innovative Deduction Learning (DL), instead of the conventional Induction Learning (IL). The domain theory of our deductive method, DL, made use of accumulated comparison of paired inputs leading to corrections to preceded measured blood glucose to construct our deep neural network architecture. DL method involves the use of paired adjacent rounds of finger pulsation Photoplethysmography signal recordings as the input to a convolutional-neural-network (CNN) based deep learning model. Our study reveals that CNN filters of DL model generated extra and non-uniform feature patterns than that of IL models, which suggests DL is superior to IL in terms of learning efficiency under limited training data. Among 30 diabetic patients as our recruited volunteers, DL model achieved 80% of test prediction in zone A of Clarke Error Grid (CEG) for model training with 12 rounds of data, which was 20% improvement over IL method. Furthermore, we developed an automatic screening algorithm to delete low confidence outlier predictions. With only a dozen rounds of training data, DL with automatic screening achieved a correlation coefficient (${R}_{P}$) of 0.81, an accuracy score (${R}_{A}$) of 93.5, a root mean squared error of 13.93 mg/dl, a mean absolute error of 12.07 mg/dl, and 100% predictions in zone A of CEG. The nonparametric Wilcoxon paired test on ${R}_{A}$ for DL versus IL revealed near significant difference with *p*-value 0.06. These significant improvements indicate that a very simple and precise noninvasive measurement of blood glucose concentration is achievable.

## Introduction

The successful story of AlphaGo^1^ using artificial intelligence (AI) to defeat any champion player gives a silver lining to solve the anticipated “holy grail” in diabetes mellitus (DM)^2^—the non-invasive blood glucose (NIBG) measurement. DM is a chronic condition of abnormally elevated blood glucose level (BGL), that typically leads to complications and damages to various parts of the body, and may further result in heart disease, kidney failure, blindness, and amputations^3^. Most of the currently available glucose monitors utilize invasive methods, which cause pain, discomfort, and may put patients at increased risk of spreading infectious diseases^4,5^. There are many attempts combining the big data analysis and helps of AI to develop NIBG estimation. Nevertheless, unlike the success of AlphaGo, which works because the playing rule of Go is universally identical, in the medical domain the physiological complexities and differences between individuals cannot be ignored. Thus, precision medicine has become the emerging stream for medical treatments.

Regarding NIBG, it has been extensively researched with the goal of helping diabetic patients to improve their quality of life^6–14^. Among these sensing technologies, photoplethysmography (PPG), an optical signal measurement technique based on near-infrared (NIR) transmittance or reflectance, is considered the most convenient and cost-effective choice^14–18^. Two main theories have been proposed to support the indication that NIBG prediction can be achieved by optical means. First, light absorption and reflection signals under specific wavelengths can be affected by blood glucose level (BGL)^15,17,19^. Second, pulsatile waveform varies with glucose levels due to hemodynamic factors^20,21^. The PPG signal can be used in both scenarios by applying different morphologic feature extraction and signal processing techniques, such as Fast Fourier transform (FFT)^22^, wavelet transform^17^, and Kaiser-Teager energy and spectral entropy^21^. However, currently, there is no evidence of strong correlation between optically-derived features with BGL. Recently, machine learning and deep learning models have been used in complicated optical signal analysis for NIBG prediction, including autoregressive moving average (ARMA)^23^, partial least square regression (PLSR)^24^, support vector machine (SVM)^25^, Random forest^26^, K-nearest neighbor (KNN)^27^, Bagged Trees (BT)^28^, Gaussian process regression (GPR)^29^, multiple linear regression (MLR)^30^ and artificial neural network (ANN)^31^.

Large variability in human physiology leads to an enormous discrepancy of the extracted PPG-signal features with respect to BGL. Thus, personalized modeling by tracking BGL in a period with different data collection methods has been explored to bridge the gap between physiological signal and BGL. A summarized table of comparing previous works of PPG based NIBG with personalized models and this work is illustrated in Table 1. Al-dhaheri et al.^32^ collected fifteen days of PPG signals from 10 healthy human subjects, where a linear regression model was trained from the first ten days and tested with the remaining five days of data. Rachim and Chung^17^ built a PLSR model from data of every 10 min pre-carbohydrate-rich meals and every 20 min post-meal, for a total of 120 min from 12 healthy volunteers, trained by one day and tested on the next day. Yeh et al.^33^ modeled two type-2 diabetes patients and one healthy male subject from data of every 15 min up to 2 h after regular meals, measured over a period of four weeks, in which the model was trained by one day and tested by the remaining 13 days of data. Though with promising results they reported, their prediction performance showed large variations from person to person, suggesting that either the models or the data collection methods may not be generally suitable for every subject. Furthermore, their prediction power over a long period remains unknown.

**Table 1:** Comparison of PPG based NIBG with personalized models.

References	Recruited subjects	Accuracy (zone A ratio of CEG)	Training Rounds for modeling	Time span between training and testing	Input data	Method	Age of population	
V.P. Rachim et al.^17^	12 healthy subjects	100%	~ 20	1 day	24 features from PPG	Linear partial least squares regression	Not reported	
Al-dhaheri et al.^32^	10 healthy subjects	> 90%	> 30	Not reported	PPG signal voltage	Linear regression	20–36	
Shu-jen Yeh et al.^33^	2 diabetes and 1 healthy subject	90% or less, subject dependent	3–4 day, with 15 min interval	1–13 days	Temperature-modulated reflectance signal	linear least square regression, retrieving training data for best model fitting	50–58	
This work	30 diabetic subjects	100% (with auto-screening); 80% (w/o screening)	12	20–85 days	PPG signal	Deduction Learning	42–76	

As the aforementioned personalized models collect data continuously from fasting to post-meal periods, the accompanying drastic physiological condition changes increase the level of complexity for modeling BGL from the PPG signal. Therefore, we focused on tracking fasting BGL to reduce the interference, which is also the preferred clinical index for the management of hyperglycemia in DM patients^34^. Since post-meal BGL tracking is excluded in our model, we extend the fasting data collection to approximately two years, focusing on the model predictability of long-term BGL variation (Supplementary Data Fig. 1).

Moreover, the aforementioned studies implemented models with the structure of conventional concurrent input–output, we hereby named the conventional modeling “Induction Learning (IL)”, which lacks the consideration of causal effect from preceded data. As illustrated in our preliminary test, in which a personalized Random Forest (RF) model was trained with limited rounds of data (see Table 3, and discussions in section “Results”), obviously it cannot attend acceptable predicting accuracy for clinical applications. In principle, IL should predict well if a lot of well annotated data sets are available for model training. However, for personalized NIBG modeling, collecting a lot of data sets is not practical, because the corresponding amount of reference BGL measured by each of individual finger-pricks for model training is quite uncomfortable and very unfriendly in daily usage. To make up the insufficient amount of training data, we turned to impose rules from our domain knowledge to guide the learning process, in order to improve performance of our model. In this work, we adopt conventional IL as a baseline for reference, and develop an innovative method, named “Deduction Learning (DL)”^35^, aiming at significantly improved prediction accuracy with only a dozen rounds of data for model training.

## Model design

**Figure 1:**
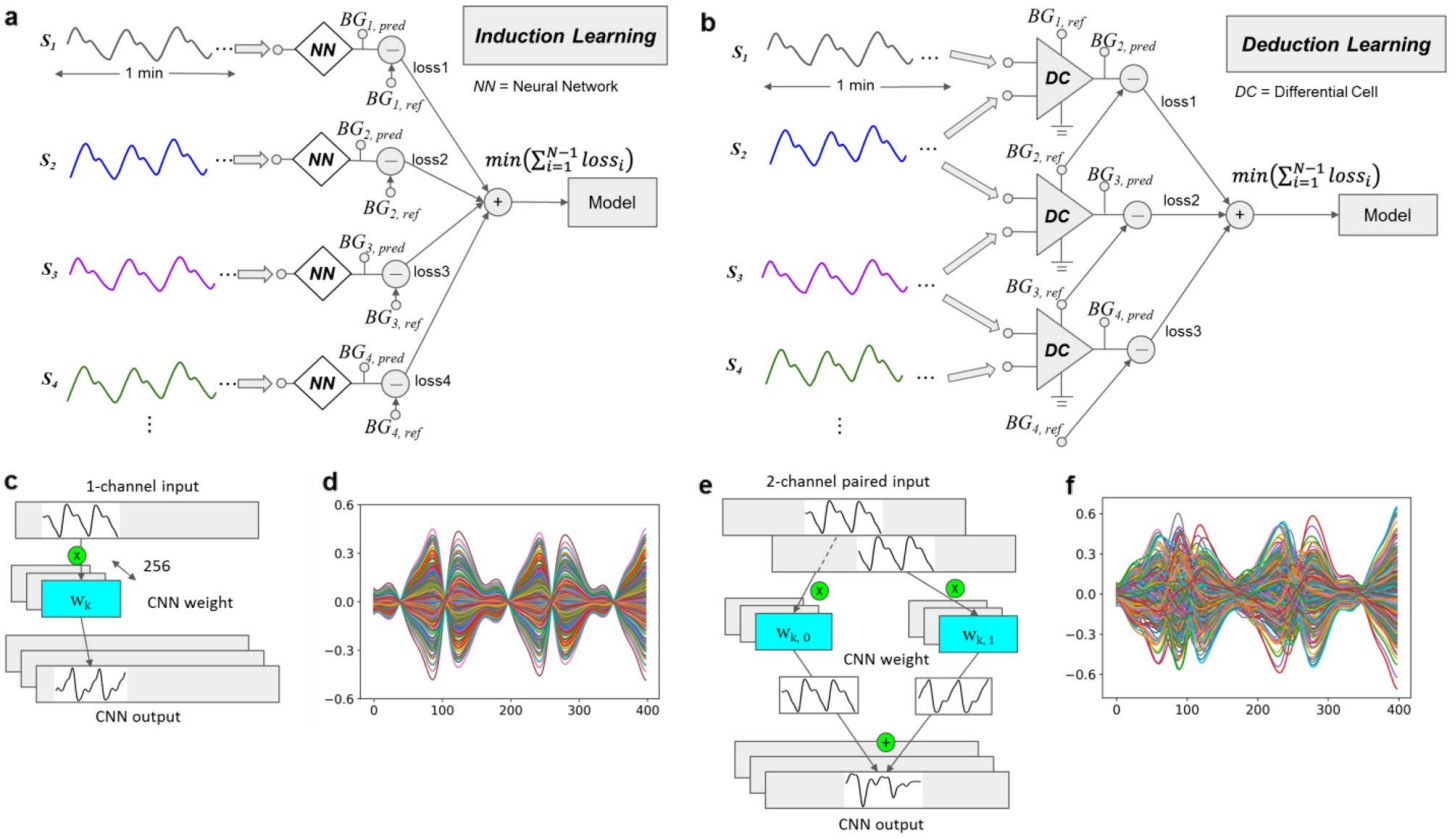
Schematic diagrams of IL and DL models. (**a**) Training of IL model. (**b**) Training of DL model. Each diamond block in (**a**) and triangular block in (**b**) represent a single personal model (NN) and a differential cell (DC), respectively, which take PPG signals as input and predicted BGL as output. Both NN and DC share similar CNN architecture (see Supplementary Data Fig. 5). The PPG signals ${S}_{1}$, ${S}_{2}$, ${S}_{3}$, ${S}_{4}$ are recorded in chronological order, with their corresponding reference glucose levels ${BG}_{1,ref}$, ${BG}_{2,ref}$, ${BG}_{3,ref}$, ${BG}_{4,ref}$. For (b), when ${S}_{i}$ and ${S}_{i-1}$ connect to the input of the DC (see Supplementary Data Fig. 2b), the loss between the reference ${BG}_{i,ref}$ and the output prediction ${BG}_{i,pred}$ will be minimized by the backpropagation of the model. (**c**) 1-channel input of signal segment convolves with a specific filter, that generates a simpler pattern of features (e.g., the reverse of the input signal). (**e**) 2-channel input generates extra and non-uniform features beyond the original signals. (**d**)**, **(**f**) One window of the overlapped output of 256 filters from the first CNN layer of IL and DL, respectively.

The schematic diagrams and pseudo codes of IL and DL models are presented in Fig. 1a,b, and Supplementary Data Code 1 and 2, respectively. In this work, both IL and DL were designed as the process of accumulated learning, with adding more and more rounds of training data. The rule imposed into our DL model is the assumption of the relation between the predicted BGL with its preceded BGL, and also the measured PPG signals:

1a $$DL \leftarrow \sum_{i=2}^{N}f\left({S}_{i} ,{S}_{i-1},{BG}_{i-1}\right)$$

1b $${BG}_{k}\leftarrow DL({S}_{k}, {S}_{N}, {BG}_{N})$$

where ${BG}_{k}$ is the predicted BGL for a given measured PPG signal ${S}_{k}$ at round $k$, ${BG}_{i}$ and ${S}_{i}$ are the preceded measured BGL ($i=1\sim N$) for model training and PPG signal at round $i$ ($N<k$), $N$ is the total number of rounds of data for model training, and the function $f$ is unknown to be deduced by machine learning. In this work, we tentatively set $N=12$ for clinically acceptable predicting accuracy (see below). Plainly speaking, we aimed to implement the deduction process of accumulated comparison between two consecutively measured PPG signals that leads to successive corrections to the preceded ground truth ${BG}_{i-1}$.

In this study, personalized data (i.e., PPG and BGL) of the recruited subjects were collected in several rounds of measurements (Supplementary Data Fig. 1) for models building and testing. Our DL model of pairing input signals for BGL prediction was inspired by the scheme of a differential amplifier (DA). For a DA (Supplementary Data Fig. 2a), it takes two signals (i.e., ${S}_{in+}$ and ${S}_{in-}$) as input, reduces the potential background noise, and amplifies the difference of the two input signals for output (i.e., ${V}_{out}$) within a voltage range given by the source (i.e., ${V}_{S+}$). Analogically, our DL model consists successive sets of differential cells (DC) (Supplementary Data Fig. 2b), each takes two signals ${S}_{i}$ and ${S}_{i-1}$ (i.e., the adjacent rounds of PPG data) forming a pair of input and a reference BGL (i.e., the precedingly measured ${BG}_{i-1,ref}$) as the baseline, and outputs the predicted BGL ${BG}_{i,pred}$ based on the correlation between extracted features of the difference ${{S}_{i}-S}_{i-1}$ and the baseline ${BG}_{i-1,ref}$. If the difference of related physiological state change revealed in the difference of ${S}_{i}$ and ${S}_{i-1}$ can be quantified and associated with BGL change, it is possible to encode and learn through pairs of recordings by a deep neural network, even though the association between the raw signal and glucose concentration may be weak.

The idea of pairing data of adjacent rounds is based on the assumption that there exists a correlation between glucose variation and the PPG signal variation. Although the correlation between rounds could also be learned from traditionally separated single input (IL), the model training could be quite inefficient and may often result in overfitting. In this work, we set out to design a more efficient way to enhance model learning with limited data through the comparison of two rounds of PPG signals. Here we demonstrate the result of a signal segment passing through a specific CNN filter, to illustrate the possible reason of superior performance in DL over IL. Figure 1d,f show a typical example (a real case in round 7 of subject 1) the major difference in the first convolutional layer between 1-channel and 2-channel inputs. With simply 1-channel input that the vector convolves to a specific filter (filter length = 3), the corresponding output generates a linear combination of three adjacent input vector elements for each output data point, which is seen as a reverse pattern in our example (Fig. 1c). On the other hand, for 2-channel input vectors, they convolve separately and then are superimposed together to form the output. This step creates more possible variations of operations to the original signal, including the one shown in Fig. 1e. As a result, unlike the 1-channel input, which reveals similar pattens in each pulse, 2-channel input leads to extra and non-uniform features than the 1-channel input, i.e., more complicated local peaks and valleys in the waveforms of feature patterns, and the feature patterns of different pulses might be quite different. This might make the model easier to establish the correspondence between the features and the predicted BGL, and also to avoid overfitting in a long period of training. More evidence of model with 2-channel input results in a more complicated structure of features than with 1-channel input is illustrated in Fig. 1d,f, and in Supplementary Data Fig. 10 for more of our tested subjects, which show the output of the first CNN layer of all 256 filters for both cases. In these examples, each 1.6 s segment contains about two pulses of PPG signal. For the case of similarly repeated two-pulse morphology in the segment, it is clear that only one pattern with positive and negative scaling comprises all the output for the model with 1-channel input; while more variations exist in the results of the model with 2-channel input. As a result, with the impose of our domain knowledge (Eq. 1, Fig. 1b), 2-channel input in DL has more potential to learn complex tasks, including the relatively weak correlation between PPG signals and BGLs. For the details of composing 2-channel input for our model, please refer to the section “Method”.

Considering clinical application, although DL was demonstrated to significantly outperformed IL in prediction accuracy (see section “Results”), a screening algorithm is needed to further exclude outliers arising from abnormal measurement of PPG signals. Our implementation of screening algorithm is illustrated in Supplementary Data Fig. 6 and 7, together with the pseudo code presented in Supplementary Data Code 3, in which two stages of screening process are implemented based on the test of validation confidence score ${S}_{V}$ and the test spread score ${S}_{T}$, respectively (see section “Method” for the definitions of ${S}_{V}$ and ${S}_{T}$). In the first stage, ${S}_{V}$ is used to examine quality of the model built from the training data of all the preceded rounds. The model quality is highly sensitive to the amount and quality of the training data. If the model cannot pass the screening of the first stage, it usually means that more rounds of PPG measurements, together with the corresponding reference BGL from finger-pricks, are needed for model building. In the second stage of screening, ${S}_{T}$ calculated from the model prediction of the testing data is checked to filter out possible outliers. This usually due to an inferior measurement of PPG signal, and redo the measurement more rigorously is usually necessary.

The threshold values of ${S}_{V}$ and ${S}_{T}$ for pass / reject decision of the built model and predictions were determined by the empirical tests and the receiver operating characteristic (ROC) curve^36^ (see Supplementary Data Fig. 8), respectively. For a given model with accepted quality (i.e., the first screening stage is passed), ROC curve provides a systematic way to search for an optimal threshold value of ${S}_{T}$ to filter out abnormal predictions. Ideally, the optimal threshold should be found near the convex point close to the upper-left corner of ROC curve plot. If the threshold is shifted along the curve to the upper right side, the condition is less stringent and more samples are accepted. If it is shifted towards the lower left side, the condition is stricter and fewer samples are included. Supplementary Data Fig. 8 shows the ROC curve calculated from all subjects after eight rounds of measurements for our models. It is interesting to compare the effect of ${S}_{T}$ screening on both DL and IL, which reveals the superiority of DL over IL more clearly. The optimal threshold values of ${S}_{T}$ for both cases are near 0.07, with the corresponding locations in ROC curve illustrated by the red dots. With this threshold value, the true positive rates of DL and IL are 78.3% and 50.8% (Supplementary Data Fig. 8a), and the reject ratios are 31.1% and 56.5% (Supplementary Data Fig. 8b), respectively. Furthermore, the ROC curve of IL is closer to the diagonal line, which indicates that the true positive rate and the false positive rate are simultaneously growing with respect to relaxing threshold. In this case, optimization of the threshold value would not improve the pass / reject accuracy significantly. On the contrary, the ROC curve of DL shows a promising shape above the diagonal line, which indicates the spread score ${S}_{T}$ is more effective to distinguish the normal and abnormal test predictions in the DL model. Therefore, we conclude that pairing of adjacent rounds of data in DL helps screening task and achieves an overall better performance.

## Results

In this work, our codes were developed in Python 3.6.6, with tensorflow 1.11.0, keras 2.2.4, on a platform of CUDA driver / runtime versions 10.1 / 9.0, and cuDNN 7.3.1.20. All the model training and testing were performed on an ASUS ESC8000 server with dual Intel Xeon Silver 4114 CPUs, 6 GPU cards of GTX 1080Ti (11 GB GPU on board memory), and 256 GB host memory. The training of the CNN architecture (Supplementary Data Figs. 2 and 5) was performed in GPUs, and the required training time and GPU resources are summarized in Supplementary Data Table 4. The column “training rounds” means training from round 1 to the listed rounds. Note that our architecture of both IL and DL are accumulated training, for example, training up to round 4 of DL involves three DC trainings of pairs $({S}_{1}, {S}_{2})$, $({S}_{2}, {S}_{3})$, and $({S}_{3}, {S}_{4})$ (see Fig. 1). To speed up the trainings as much as possible, in each case we performed all the involved NN (for IL) or DC (for DL) trainings parallelly. Thus, for trainings up to more rounds, the required GPU resources grew roughly linearly.

**Figure 2:**
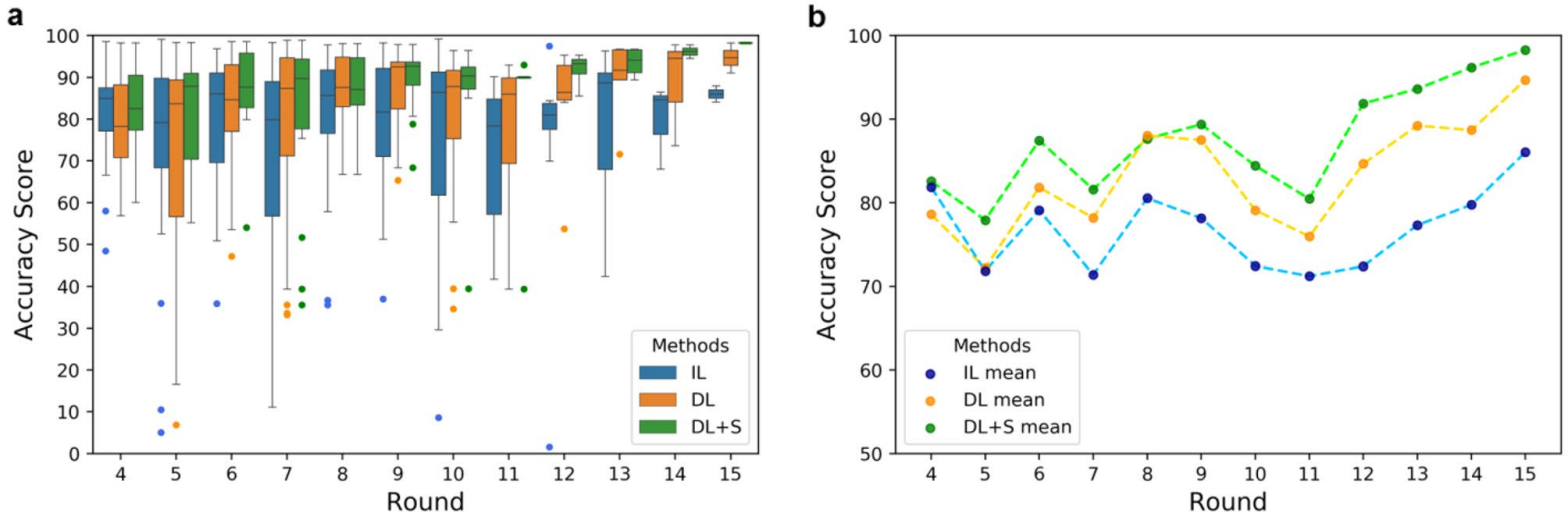
Comparison of accuracy scores (*R*_*A*_) of each method on all 30 subjects in each test round. (**a**) The accuracy score distribution in a box plot. (**b**) The mean accuracy scores in a line chart.

To present the prediction results in a timeline scenario, in Fig. 2a,b we show the average and variation of predicting accuracy score of all subjects for every test round since round 4. In this study, the accuracy score is defined in Eq. (4), which is the relative difference between the predicted BGL and the ground truth. Here (and also all the following results unless described specifically), the result of round $i$ represents the prediction of PPG signal samples of paired (for DL and DL + S) data from round $i$ and round $i-1$, and non-paired (for IL) data from round $i$, respectively, by the corresponding models built with the training data collected in the preceded rounds from round 1 to round $i-1$. Evidently, marked improvement of the mean accuracy scores is found between DL (orange line) and IL (blue line). Furthermore, the variation of prediction accuracy of IL is much larger than that of DL. This result strongly suggests that DL model outperforms IL model both in prediction accuracy and stability. This enhancement is due to the influence of data pairing. In addition, with the screening enabled, the mean accuracy score of DL + S (DL with screening, green line) is further improved to more than 90 after round 11. This demonstrates that our screening mechanism works prominently in filtering out the bad predictions.

Due to the reduced number of subjects in the later rounds (see Supplementary Data Table 2), the averaged accuracy of each round may not be representative to reveal the true statistics. In order to disclose the real trend of prediction accuracy in the long term, in Table 2, the overall performance of three models, IL, DL, and DL + S are presented in groups of rounds 4–7, 8–11, 12–15, and the total (4–15). More detailed data can be found in Supplementary Data Table 3. Comparing IL and DL, it is clear that, unlike DL, both the prediction accuracy and Pearson correlation coefficient do not improve with increasing rounds of training data. There are fluctuations, which lead to an overall almost flattened trend in accuracy score but actually decreasing correlation with more accumulated training data. This seems to be a common phenomenon of the traditional IL, that long-term NIBG prediction from PPG signals may gradually fail, and it seems to be helpless with more training data added. On the other hand, DL has significant improvement in prediction with more and more training data accumulated. This exhibits a remarkable difference between IL and DL. Furthermore, enabling screening to separate out the bad predictions, both the accuracy and the correlation gain more improvement of > 0.06 and significantly > 0.17 over DL, respectively, in the group of rounds 12–15.

**Table 2:** Performance Summary of models in groups of rounds.

Model	Ratio in zone A of CEG		Accuracy score (*R*_*A*_)				Pearson correlation coefficient (*R*_*p*_)			
Rounds	8–11	12–15	4–15	4–7	8–11	12–15	4–15	4–7	8–11	12–15
IL	58.2%	61.1%	76.29	76.11	76.54	76.50	0.554	0.578	0.540	0.417
DL	76.9%	80.6%	80.62	77.68	83.90	87.70	0.640	0.606	0.674	0.782
DL + S	85.1%	100%	84.70	81.90	86.84	93.87	0.725	0.646	0.812	0.960

**Figure 3:**
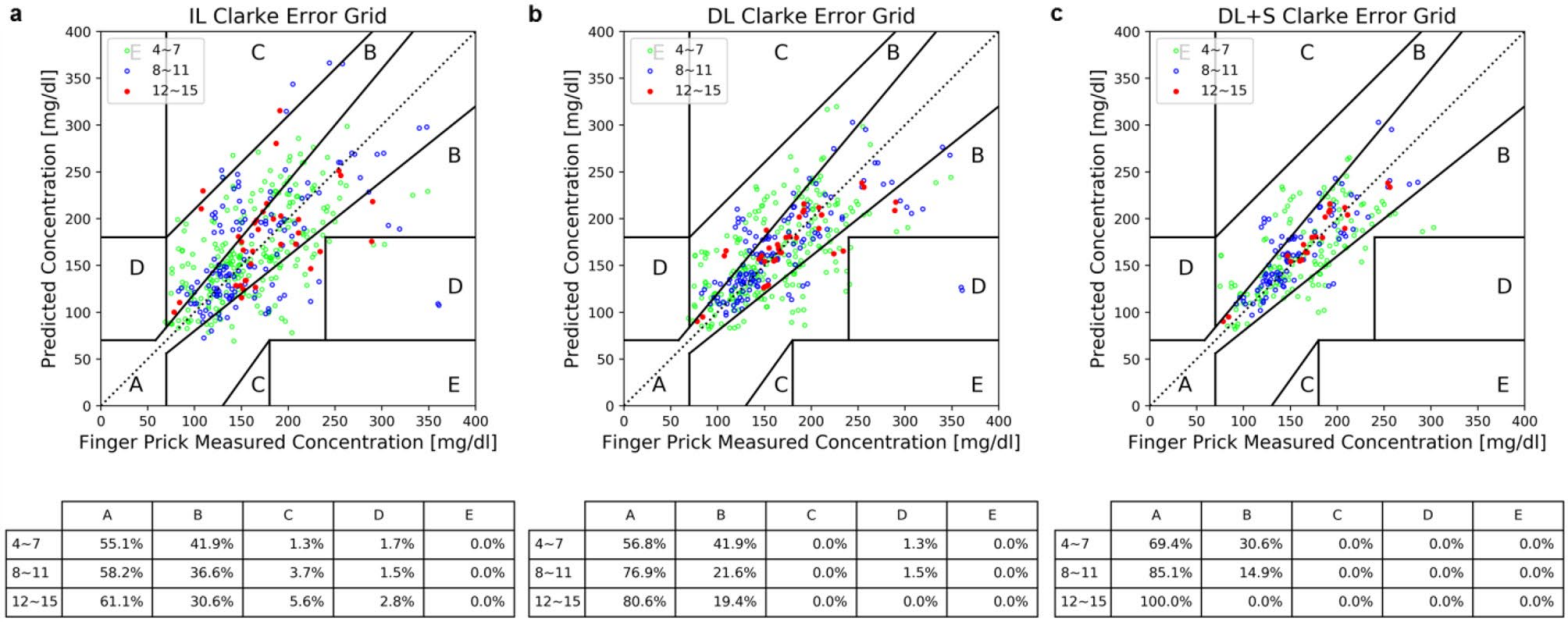
Clarke Error Grid (CEG) plots of model predictions: (**a**) IL. (**b**) DL. (**c**) DL + S. The data points are grouped into three categories: green symbols are results of 4th to 7th rounds, blue symbols are results of 8th to 11th rounds, and red symbols are results of 12th to 15th rounds, respectively. The table below each figure lists the proportion of data points found in each zone of the CEG. For DL and DL + S, all data points of rounds 12 to 15 are found in zone A and B.

The difference between the predicted BGL versus the reference BGL can be more clearly visualized by Clarke Error Grid (CEG) plots^37^. Comparing IL and DL, as shown in Fig. 3a,b, the predicted data points in zone A are significantly increased in rounds 8–11 and rounds 12–15 of DL, about 19% and 20% improvements over IL in predictions in zone A of CEG, except the particular outlier point (blue, at round 10) in zone D of IL and DL. Examining this outlier more closely, it possesses a high reference BGL (> 350 mg/dl), higher than BGLs in the preceded rounds utilized to train the model. Nevertheless, with screening enabled (Fig. 3c), this outlier is removed. As a result, with DL + S, it not only gives enhanced prediction accuracy, but also delivers a more robust result without the erroneous and misleading predictions.

**Figure 4:**
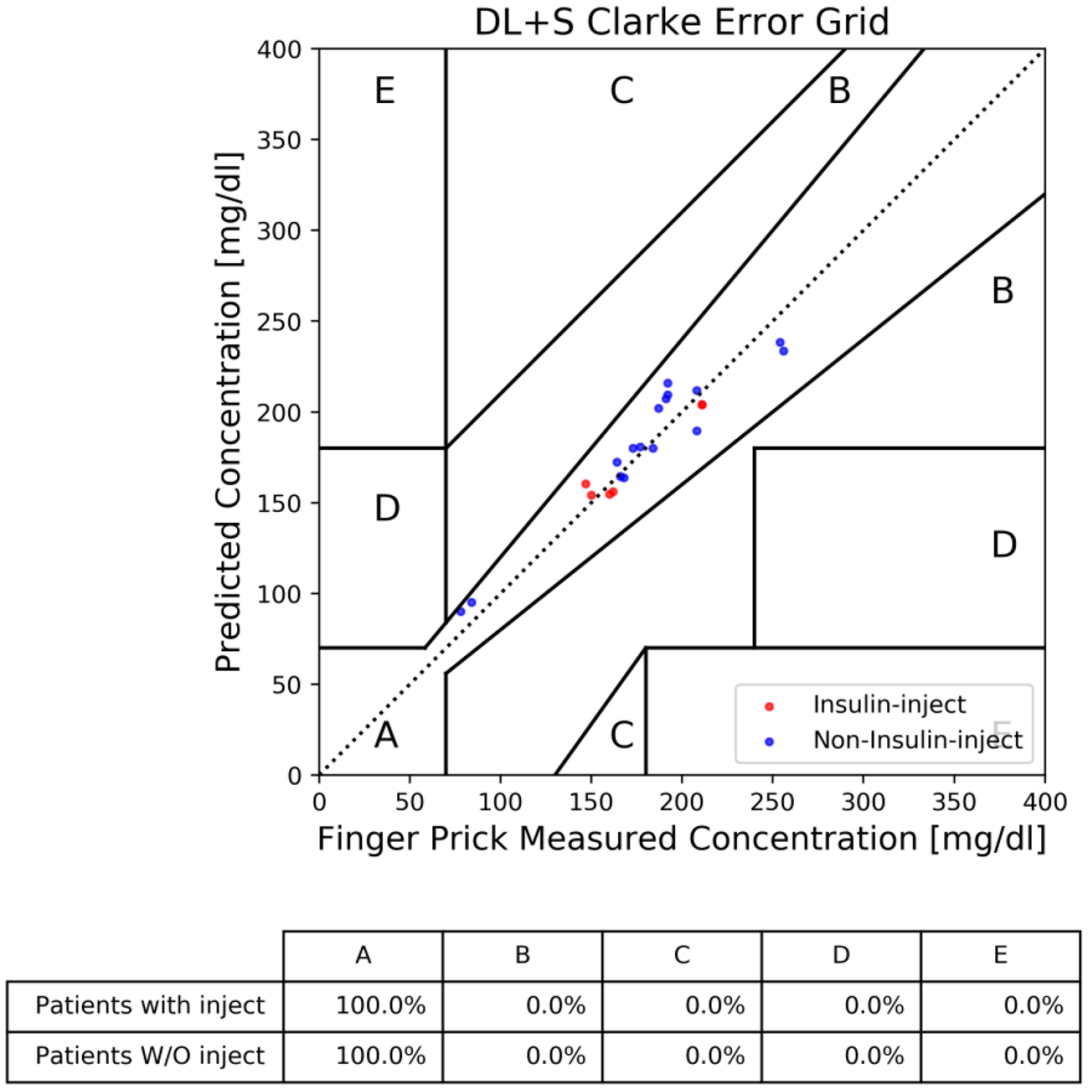
Comparison of DL + S predictions for patients with and without insulin treatment at rounds 12–15.

The influence of insulin injection on the accuracy of BGL prediction was also investigated. As illustrated in Fig. 4, stratification of DM patients into with / without insulin injection groups illustrates a similar pattern in the CEG plot. This demonstrates the uniformity of DL + S model prediction on DM patients regardless medical treatment.

**Figure 5:**
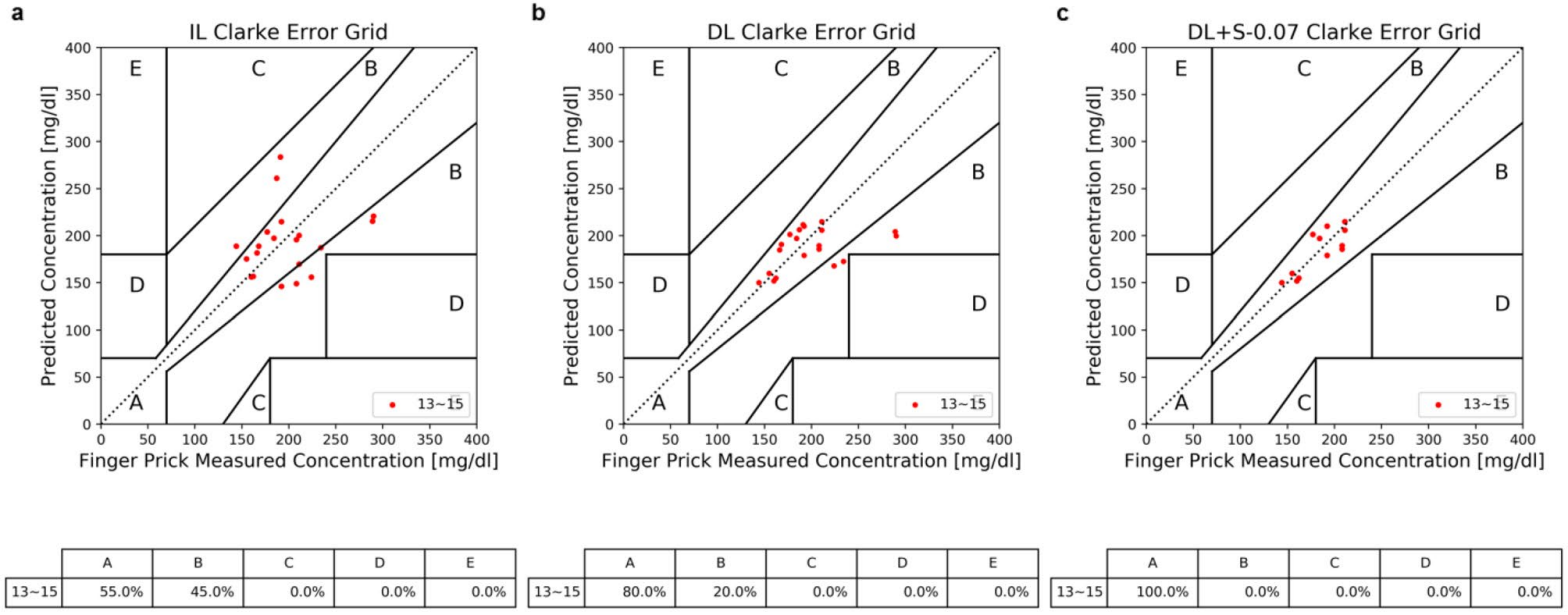
Predictions of rounds 13–15 (each paired with round 12) by models built with a dozen rounds of training data (rounds 1–12) for (a) IL, (b) DL, and (c) DL + S.

Finally, we test a scenario of a real application. For BGL estimation based on a personalized model, the first step is training the model with enough personal data, together with the corresponding real BGL measurements from finger-pricks, and then uses the model for forthcoming predictions. A clinically useful model should be able to preserve prediction quality for a reasonably long period, without adding more training data to regulate the model from deviations. In this test, the models were built with a dozen rounds of training data (rounds 1–12), and then were tested in rounds 13–15. The data pairing for running this test is data of round 12 paired with data of rounds 13–15 to predict BGLs for rounds 13–15, respectively. The prediction results are presented in Table 3, Fig. 5, and Supplementary Data Fig. 11. Here, to be completeness, we also incorporate results of our preliminary tests of Random Forest (RF) model, to illustrate the general property of performance difference between IL and DL.

**Table 3:** Performance of models with 1st to 12th rounds as training and rounds 13, 14, 15 as testing (each paired with round 12).

Methods	DL + S	DL	IL	RF w/o signal	RF w/ signal
Accuracy score (${R}_{A}$)	93.50 $\pm$ 3.50	88.21 $\pm$ 8.82	81.47 $\pm$ 12.28	80.46 $\pm$ 14.21	82.13 $\pm$ 12.82
Mean absolute error (MAE) [mg/dl]	12.07	25.96	38.38	40.46	37.19
Root mean squared error (RMSE) [mg/dl]	13.93	36.25	46.51	53.18	48.88
Pearson correlation coefficient (${R}_{P}$)	0.81	0.44	0.2	−0.08	−0.018
A-Zone ratio	100%	80%	55%	60%	60%

Our preliminary tests of personalized Random Forest (RF) model are also presented here. The A-Zone ratio is the ratio of data points located in the zone A of CEG plot, and ${R}_{A}$, MAE, RMSE, and ${R}_{p}$ are defined in Eqs.4, 5, 6, and 7, respectively. Since each sample has its ${R}_{A}$ value, the average and standard deviation for each model were presented.

In our preliminary tests, the RF models, which are also regarded as the framework of IL, were trained with 6 morphological features data only (labeled as “RF w/o signal”), and with both 6 morphological features and PPG signals data (labeled as “RF w/ signal”), of rounds 1–12, respectively. For IL and DL(+ S), we used both 6 morphological features and PPG signals data for model training (see section “Methods”, Data Preprocessing, and Supplementary Data Fig. 4). But for the RF models, in practice usually only distinct features are used as the training data, like the case of “RF w/o signal”. Here to be completeness and fair comparison, we also tried the case of “RF w/ signal”, i.e., using exactly the same training data as that of IL and DL(+ S) for the RF models.

Our prediction result shows the accuracy of DL + S outperformed DL, and DL outperformed IL and RF. Comparing IL and the two RF results, although they have similar ${R}_{A}$, MAE, RMSE, and A-zone ratio, but ${R}_{P}$ (Pearson correlation coefficient) of the two RF results is worse than that of IL. This is also apparent in CEG plots. Comparing Supplementary Data Fig. 11 and Fig. 5a, the data points of RF results tend to lie flat instead of lying along the diagonal line, which means that it is more difficult for RF to figure out the correlation of input data and BGL, and thus produced worse predictions. Next, comparing IL and DL(+ S), almost half of predicted data points reside in zone B of CEG plot for IL, and for DL and DL + S, the predicted data points in zone B are reduced to 20%, and completely removed with the help of screening, respectively. All the other metrics also show the performance enhancement of DL(+ S) over IL. In other words, DL gives more promising predictions than IL, and DL + S ensures the confidence of predictions in minimal obscurities.

Finally, in order to objectively compare the performance between DL and the models of IL and RF models individually with quantified metrics, the nonparametric Wilcoxon^38^ paired test of these predicting results was conducted. Among the performance metrics listed in Table 3, only ${R}_{A}$ has a data population in each model, hence ${R}_{A}$ was used to perform the paired test. Here we were aiming to compare the direct predictions of DL with IL and RF models specifically, thus only the paired tests of DL with these models were conducted separately. Further, the tests of DL + S with the other models were skipped, because DL + S essentially gave the same predictions as DL, except that some predicted data considered as outliers were removed by screening. The Wilcoxon paired test results of ${R}_{A}$ gives *p*-value = 0.063 for DL versus IL, 0.068 for DL versus RF w/o signal, and 0.165 for DL versus RF w/ signal, respectively. These p-values were just near significant (0.05) due to some overlaps of their ${R}_{A}$ distributions (as indicated in standard deviations of ${R}_{A}$, see Table 3). But from viewpoint of clinical criteria, such as ${R}_{p}$ and A-zone ratio, DL significantly overwhelmed IL and RF models by more than 20% improvement. As a result, we conclude that DL(+ S) is significantly outperformed IL and RF for non-invasive BGL prediction.

## Conclusion

To solve the problem of inaccurate personalized NIBG (noninvasive blood glucose) prediction due to insufficient amount of training data, a novel prediction method, Deduction Learning (DL), is presented. The DL method involves the use of paired adjacent rounds of finger pulsation Photoplethysmography (PPG) signal recordings as the input to a convolutional-neural-network (CNN) based deep learning model. It reliably predicts fasting blood glucose level (BGL) of diabetes mellitus (DM) patients, either with or without insulin injections. For subjects with 12 rounds of data for model training and tested with rounds 13–15, DL + S achieved an accuracy score of 93.50, a root mean squared error (RMSE) of 13.93 mg/dl, and a mean absolute error (MAE) of 12.07 mg/dl, in which the improvement in accuracy over the conventional method is more than 12%. The paired t-test on MAE and (${R}_{A}$) of DL with respect to IL also revealed highly significant in predicting power, with *p*-values smaller than 0.04 and 0.03, respectively. Furthermore, DL + S attended 100% of prediction data in zone A of CEG plot. This significant enhancement might be attributed to more feature patterns arising from CNN process with the pairing mechanism. It emphasized the differentiation between two contiguous PPG records, which is the direct consequence of imposing (Eq. 1, Fig. 1b) in our model design to guide the learning, that could be helpful for CNN to find the concealed correlation between the PPG signal and BGL. It also indicates that a very simple and precise noninvasive measurement of BGL is achievable. Given more amount and types of subject-specific data accumulated, it would be promising not only to enhance the prediction quality of DL, but also to explore more possibilities in biomedical and clinical applications.

Moreover, with imposing the rule (Eq. 1, Fig. 1b) in DL model design, the guided learning process demonstrated a prominent example of deduction learning implementation. We hope it could also contribute to more innovative ideas of the model design for other data insufficient and realistic applications with machine learning.

## Methods

### Sample source

Six to fifteen recurring rounds of PPG signal measurements and invasive fasting glucose recordings from 30 volunteers were taken. All subjects were fully informed and written consents were obtained from all subjects for the collection of data and its uses. Since fasting BGL is relatively stable within a period of time, we have set the duration of our sampling intervals to range from days to months in order to collect sufficient amounts of variations in BGL (Supplementary Data Fig. 1). The collection of samples in this study has been approved by the Institutional Review Board of Academia Sinica, Taiwan (Application No: AS-IRB01-16081) and this study were performed in accordance with the relevant guidelines and regulations. Test subjects are all DM patients, of which eight took insulin treatment and others did not, as shown in Supplementary Data Table 1. Several rounds of measurements were collected from these test subjects, as summarized in Supplementary Data Table 2. Measurements of PPG signals and invasive glucose values were taken using the TI AFE4490 Integrated Analog Front End and Roche Accu-check mobile, respectively. Experiments were conducted inside the laboratory with a standard protocol including basic physical check-up, a questionnaire, and two replicates of 1-min PPG signal measurements, each with a corresponding reference BGL measurement. The detailed experimental setup and procedures can be found in^39^.

### Comparison of models

The complete workflow of our DL + S model contains PPG signal segmentation, feature extraction from PPG signal waveforms, data pairing, modeling, and screening, in which data pairing and screening are not presented in traditional IL model. To evaluate the importance of these key steps on the influence of model prediction power, the following three cases are compared.

1. Induction Learning (IL): With one channel preprocessed signal as input of the CNN architecture, the accumulated data is trained with the traditional form^14–17^. Measurement data from the present round gives the prediction directly.
2. Deduction Learning (DL): Pairing of adjacent rounds of measurements as a two-channel input, together with BGL measured in the preceded round of the paired input as the reference, to the CNN architecture.
3. Deduction Learning with screening enabled (DL + S).

In all the models, the preprocessing of data of each round, the CNN architecture, and the procedures of model training, validation, and testing are all the same, as described detailly in the following.

### Data preprocessing

A typical PPG waveform measured from the subjects is illustrated in Supplementary Data Fig. 3. The raw signal reveals pulses in varied amplitudes (Supplementary Data Fig. 3a), each pulse corresponds to a single heartbeat. The raw signal can be separated into the low frequency part (Supplementary Data Fig. 3b) and the high frequency part (Supplementary Data Fig. 3c) by a Butterworth filter^40^ with the cutoff frequency 0.75 Hz. The high frequency part is used for the following feature extraction and model input. The valleys and peaks of the high frequency part is automatically annotated by Bigger-Fall-Side algorithm^41^. The idea comes from observation of the pulses’ waveform, i.e., every peak is immediately followed by a valley with the largest magnitude difference in the pulse, which we called the “bigger-fall-side-slope” (BFSS). Since most people have 60–90 heartbeats within one minute, we sorted all the available slopes between nearby local minimum and local maximum in descending order, and selected the 30th one as the medium of BFSS values (mBFSS). All the slopes fallen into the range [0.5, 1.5] of mBFSS were identified as BFSS. Thus, the peaks and valleys of the whole waveform can be correctly labeled.

By annotating the valleys of the waveform, a PPG signal segment (which we called a window) is extracted from each valley backwardly to 400 data points earlier, which covers 1.6 s that include at least one pulse of the PPG waveform (Supplementary Data Fig. 4a). For a subject with heart rate of 60 beats/min, 60 windows are generated. Six morphological features including heart rate, area under the curve of the waveform, full width and width at 25%, 50%, 75% of maximum peak amplitude (FW_25, FW_50, FW_75) are extracted from the corresponding window (Supplementary Data Fig. 4b). The extracted features are then concatenated with the data of signal segment to form a vector with 406 elements. Thus, a round of measurement with two replicates generates data arrays with size $\left({N}_{i1},406\right)$ and $({N}_{i2},406)$, respectively, where ${N}_{i1}$ and ${N}_{i2}$ represents the number of windows of the two replicates in the round $i$.

Here comes the difference between IL and DL(+ S) models. In preparing a sample input data of the replicate $k$ ($k=1, 2$) in round $i$ to the CNN architecture, data of window ${j}_{k}$ (which is a vector containing 406 elements) is sampled out to form a 1-channel input for IL; while for DL(+ S), the window ${j}_{k}$ in replicate $k$ of round *i*th is paired with a randomly selected window ${{j}^{^{\prime}}}_{k}$ from the same replicate $k$ of the preceded round $i-1$ to form an input sample labeled $(i,{j}_{k})$ (Supplementary Data Fig. 4c). In our work, pairing involves the combination of samples between two consecutive rounds of measurement, which may be in a time span of days or months, depending on our accumulation of data from subjects. For both IL and DL(+ S), the total number of input samples of round $i$ is ${N}_{i1}$ and ${N}_{i2}$ for the two replicates, because of the ${N}_{i1}$ and ${N}_{i2}$ windows available in two replicates of measurement in this round.

### The model

The detailed layout of our CNN architecture is illustrated in Supplementary Data Fig. 5. For DL(+ S), the samples with paired windows are designed as two channels for model input, each channel corresponds to one of the paired windows. The input data is then passed through five units of CNN layers with number of filters 256, 256, 512, 1024, and 2048. Our tests shown that more CNN layers may potentially give higher predicting accuracy. Since each CNN layer consists of a maxpooling layer with pool size 2, the data vector reduced half when passing through each layer, thus it restricted the number of CNN layers one can construct. The choice of number of filters in each CNN layer is a balance of trying to extract as more features as possible from input data, and controlling the total amount of trainable parameters in the model. With our setting of number of filters, the total amount of trainable parameters is about 100,800,000, which is manageable in our GPU computing platform. The learning curves up to 1000 training epochs of our models (IL and DL) are presented in Supplementary Data Fig. 9, which shows no overfitting since the curves of testing loss decreased together with the training loss. Due to the limit of the GPU memory, the batch size is set to 3000. Finally, ReLU (Rectified linear units), Adam (Adaptive Moment Estimation), and mean squared error were adopted as our activation function, optimizer, and loss function in our model, which just followed common practice of CNN development. After the five layers of CNN, the flattened output is merged with BGL of the preceded round ${BG}_{{i}_{k}-1,ref}$ (for replicate $k$), and then go to two fully connected layers after batch normalization to get the BGL prediction ${BG}_{{i}_{k},pred}$. On the other hand, IL shares the same architecture except that there is only one channel for model input, as information of the preceded round is not considered.

### Training, validation, testing, and screening

The model training, validation, and testing processes rely on a well-defined data splitting configuration. Supplementary Data Fig. 6 and 7 illustrates an example of taking data of round 5 as a prediction test. For the training and validation processes shown in Supplementary Data Fig. 6, the standard “leave-one-out” cross-validation procedure was performed on data of all the preceded 1st–4th rounds to examine whether the model is overfitted. In addition, for DL + S that screening is enabled, we define a validation confidence score (${S}_{V}$) based on the scattered location of predicted BGLs in CEG plot analysis. By counting the accumulated validation data points inside each zone of the CEG plot, we define:

2 $${S}_{V}=\sum_{i\in U}{w}_{i}{C}_{i}/\sum_{i\in U}{C}_{i}$$

where $U$ stands for the union of all zones (from zone A to zone E), ${w}_{i}$ is the weight of zone $i$, and ${C}_{i}$ is the number of points inside zone $i$. When $i$=A, ${w}_{A}$=1; when $i$ =B, ${w}_{B}$=0.5; and when $i$=C, D, and E, ${w}_{C}={w}_{D}={w}_{E}=0$. In other words, only data points in zone A and zone B receive non-zero weights, because these predictions are relatively acceptable BGL measurements clinically. During validation, the more predicted data found in zones A and B, the higher ${S}_{V}$_._ After calculating ${S}_{V}$ from all the cross-validation data, total ${S}_{V}$ was evaluated. If this score is lower than a pre-determined validation threshold, the process stops and rejects this model training as well as the following test prediction. The threshold value depends on the number of accumulated rounds of data, which is empirically set to 50 if test round is < 7 and 60 if test round is ≥ 7. This is the first stage of the whole screening process, which is used to examine the quality of the trained model based on data of all the past rounds.

After the task of cross-validation, all the preceded data (i.e., data of rounds 1–4 in this example) were used to train the final model, and the present data (i.e., data of round 5 in this example) was used to test the model, as shown in Supplementary Data Fig. 7. For DL + S, the final model was repeatedly trained $N$ times, each with the same set of training data, but with different random number sequences, and tested by the same testing data. Thus, an additional screening procedure was carried out by the test spread score ${S}_{T}$, which is defined as the variation of the predicted BGLs ${BG}_{pred}^{(i)}$, $i=1, 2, \dots N,$ by the $N$ repeatedly trained models, with the maximum and minimum predicted values excluded:

3 $${S}_{T}=\sqrt{\frac{\sum_{i\in \mathrm{n}}{({BG}_{pred}^{(i)}-{\overline{BG} }_{pred})}^{2}}{N-1}}/\mathrm{Median}\left({BG}_{pred}\right),$$

where set $\mathrm{n}=\{1, 2, \dots N$} contains the repeatedly trained *N* rounds and $|n|=N-2$ due to removing of the maximum and minimum outcomes. To do the screening, ${S}_{T}$ must be smaller than a threshold value $y$, then the median value of all the ${BG}_{pred}^{(i)}$ is accepted as the final prediction. This is the second stage of the whole screening process, which is to remove the abnormal outliers of the prediction. The optimal value of the threshold $y$ was systematically determined by the ROC curve^36^. In Supplementary Data Fig. 8, the true/false positive rate of model predictions was investigated with respect to tuning of the threshold value $y$, and the optimal value should be near the convex point close to the upper-left corner of the plot of ROC curve. Since there are two replicates of measurement in each round, “true” is defined by either both predictions are in zone A, or one is in zone A while the other one is in zone B, of the CEG plot.

### Performance evaluation

The performance metrics of glucose prediction in test samples are calculated by the accuracy score (${R}_{A}$), mean absolute error (MAE), root mean squared error (RMSE), and the Pearson correlation coefficient (${R}_{p}$). They are defined as follows:

4 $${R}_{A,i}= \left|\frac{{BG}_{pred,i}-{BG}_{ref,i}}{{BG}_{ref,i}}-1\right|\times 100$$

5 $$\mathrm{MAE}=\frac{1}{n}{\sum }_{i}\left|{BG}_{pred,i}-{BG}_{ref,i}\right|$$

6 $$\mathrm{RMSE}= \sqrt{{\sum }_{i}({BG}_{pred,i}-{BG}_{ref,i}{)}^{2}/n}$$

7 $${R}_{p}= \frac{\sum_{i}\left({BG}_{ref,i}-\overline{{BG}_{ref}}\right)\left({BG}_{pred, i}-\overline{{BG}_{pred}}\right)}{\sqrt{\sum_{i}{{BG}_{ref,i}}^{2}-{\left(\sum_{i}{BG}_{ref,i}\right)}^{2}}\sqrt{\sum_{i}{{BG}_{pred,i}}^{2}-{\left(\sum_{i}{BG}_{pred,i}\right)}^{2}}}$$

where $i$ is the index of each samples, with $i=1, 2, \dots , n.$

## Supplementary Information

Supplementary Information.

## Data Availability

Our codes are available in https://github.com/jackielu4119/DL.
