# Supplementary material for: Deduction learning for precise noninvasive measurements of blood glucose with a dozen rounds of data for model training
Source: Sci Rep. 2022 Apr 20;12:6506. doi: 10.1038/s41598-022-10360-3 (PMC9021306; doi:10.1038/s41598-022-10360-3)
Supplement: Supplementary file 1 — Supplementary Information. [file 41598_2022_10360_MOESM1_ESM.docx]

**Supplementary Data**

**Deduction Learning for Precise Noninvasive Measurements of Blood Glucose with a Dozen Rounds of Data for Model Training**

Authors: Wei-Ru Lu, Wen-Tse Yang, Justin Chu, Tung-Han Hsieh, Fu-Liang Yang


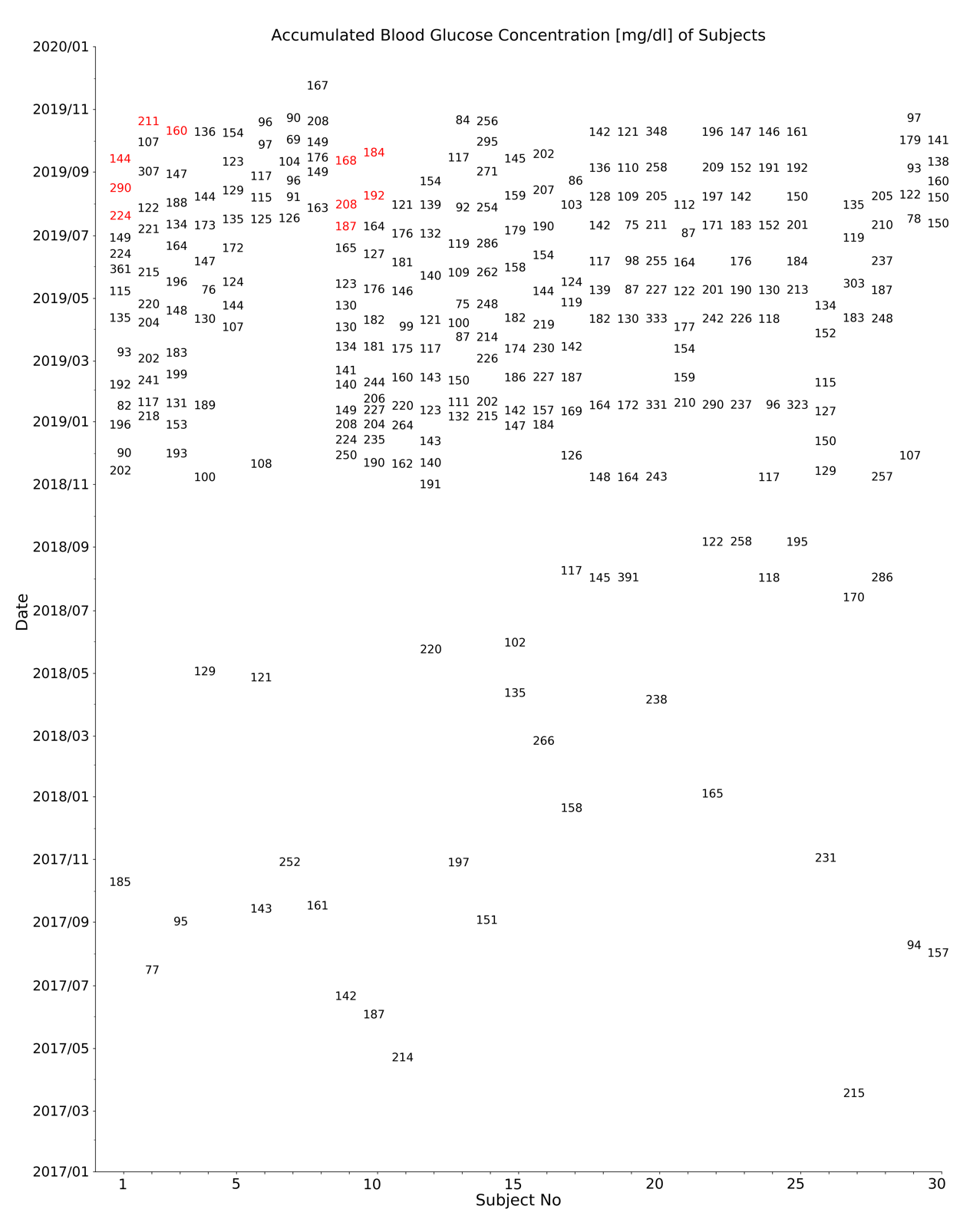


**Supplementary Data Figure 1| Accumulated Blood Glucose Concentration in Subjects.** Each of the values shown in the plot represents the mean glucose concentration (mg/dl) of one round of measurements. Samples of the same individual are vertically aligned in ascending chronological order. Red samples are the rounds of measurements after the 12^th^ round.


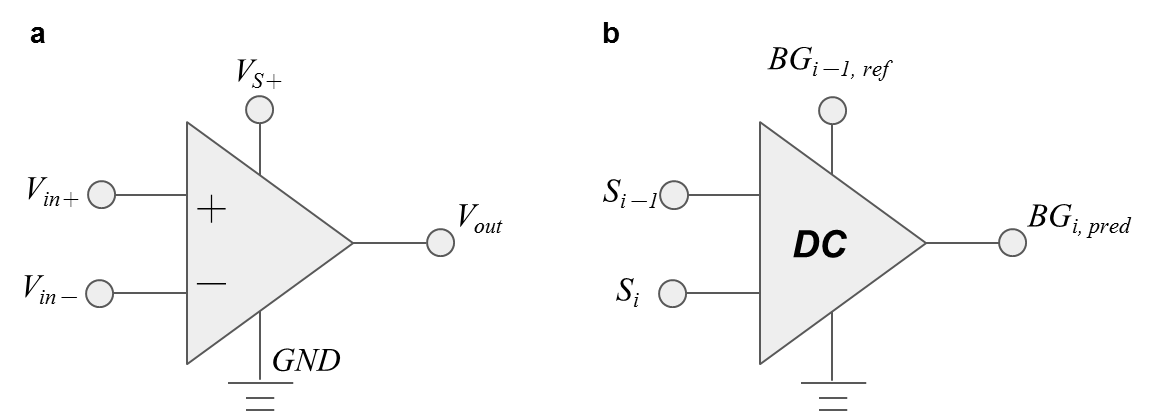


**Supplementary Data Figure 2| Differential amplifier analogy of differential cell for DL method.** (a) A differential amplifier (DA). (b) The differential cell (DC) in DL method. The CNN structure of DC is presented in Supplementary Data Figure 5.


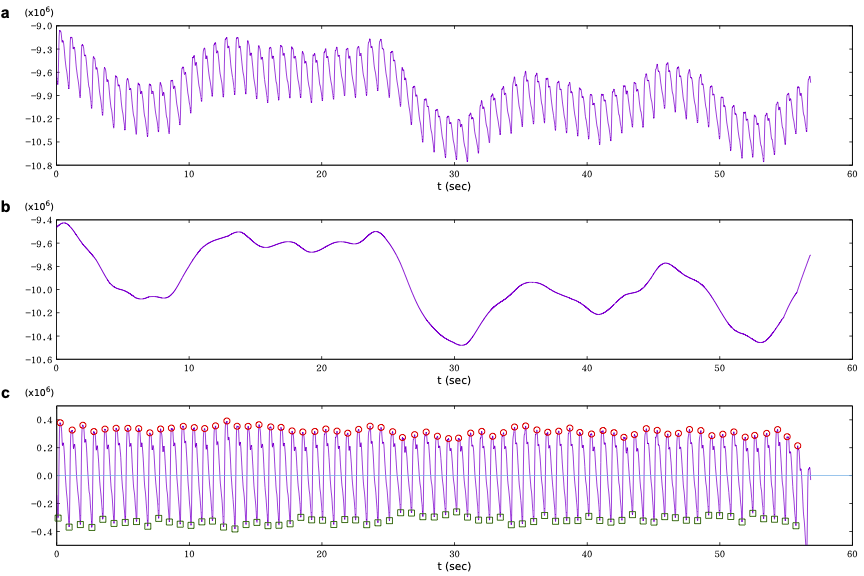


**Supplementary Data Figure 3 | A typical PPG waveform measured from the subjects. (a)** The raw signal, consisting the low frequency part and the high frequency part. (b) The low frequency part of PPG signal. (c) The high frequency part of PPG signal, in which the wave peaks are annotated by red circles, and the wave valleys are labeled by green boxes.

**a**

**
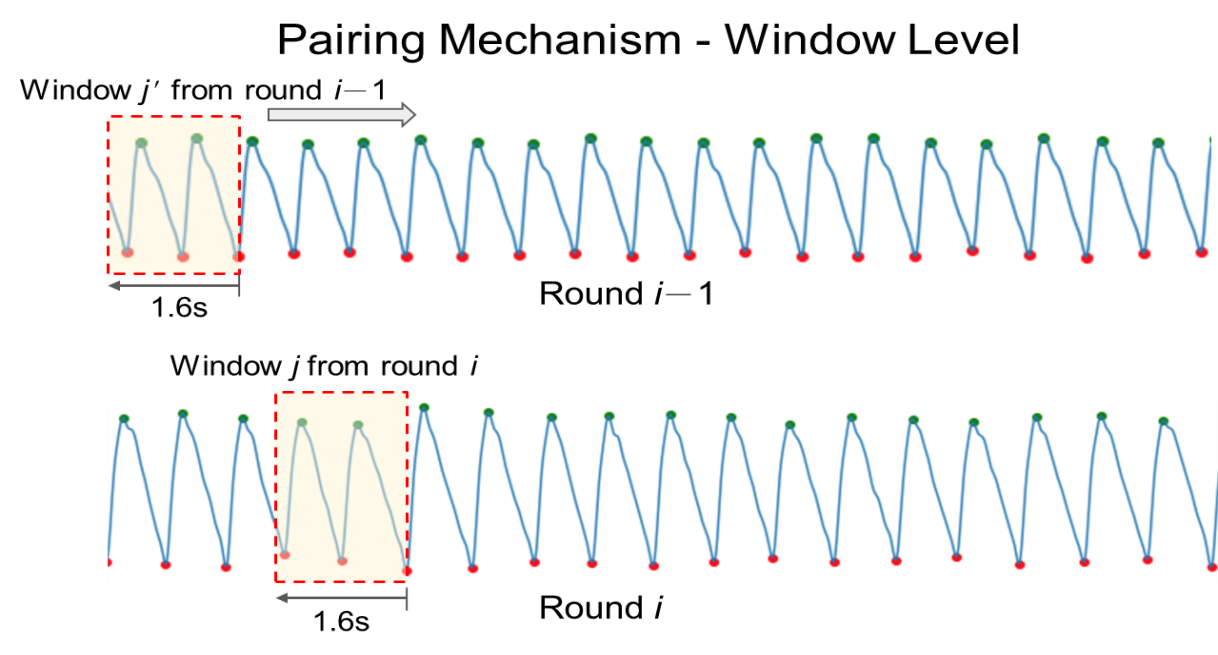
**

**b**

**
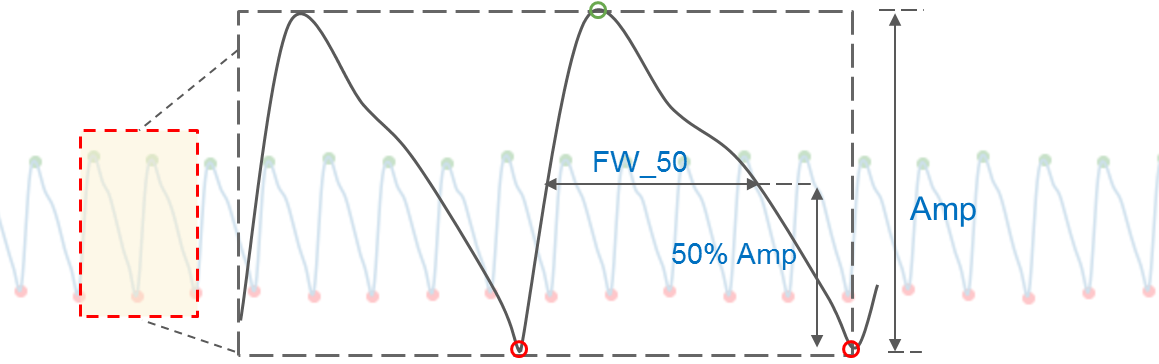
**

**c**

**
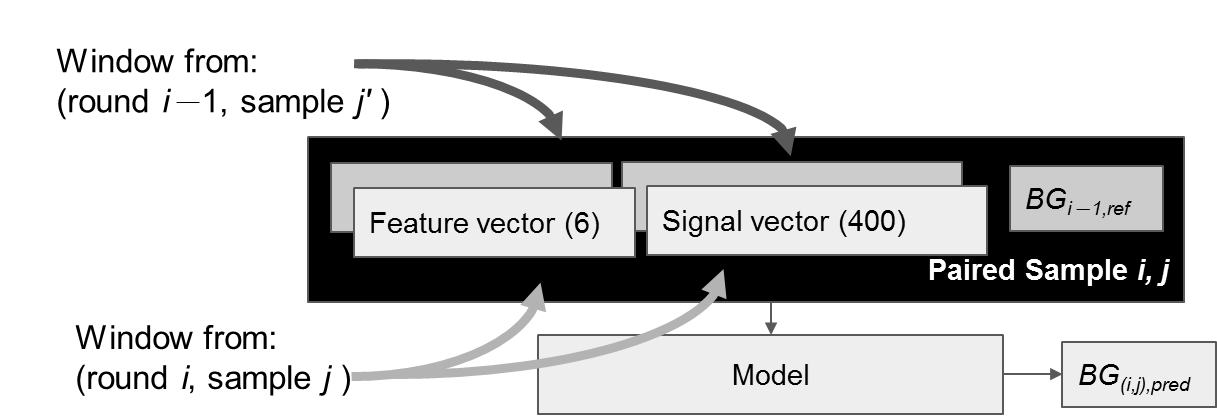
**

**Supplementary Data Figure 4| Pairing mechanism of one of the replicates in DL model. (a)** Extraction of signal segments. A signal segment (window) of a round of data is cut from each valley of the PPG waveform backwardly up to 400 points (1.6 seconds). **(b)** Three morphological features extracted from a window of signal waveform. **(c)** The data structure of one sample (labeled by $(i,j)$) of paired windows for model input. It consists of a paired data arrays from window $j$ of round $i$ and a randomly selected window $j^{'}$ of the adjacent round $i-1$, together with a reference BGL ${BG}_{i-1,ref}$. Each of the paired data arrays contains a morphological feature vector and a signal vector of the selected window. The sample $(i,j)$ yields a ${BG}_{\left( i,j \right), pred}$ by model prediction.

**
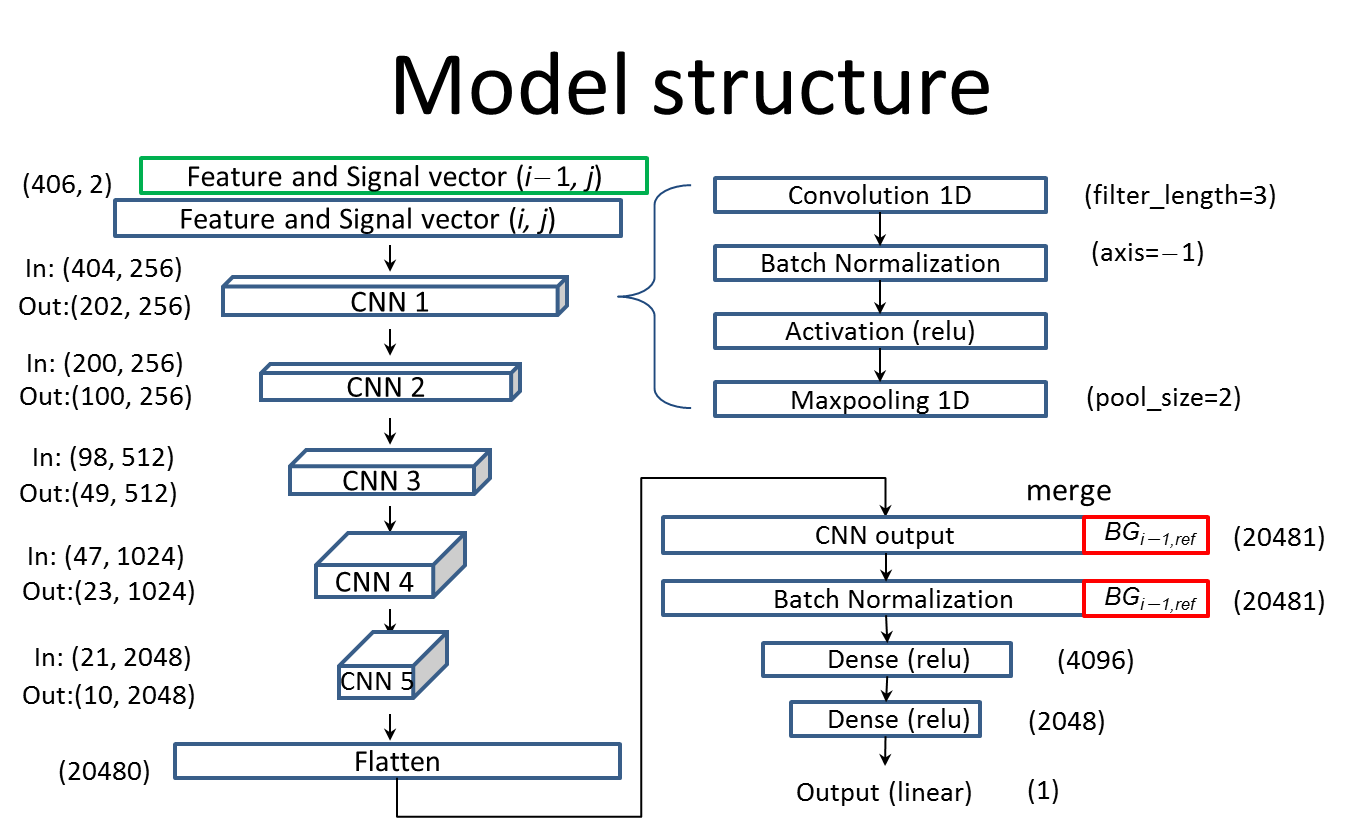
**

**Supplementary Data Figure 5 | CNN architecture of the three models.** The input array contains concatenation of PPG morphological features (6 elements) and a segment of PPG signal (400 elements), with dimension (406, 2) in pairing methods. Then it is followed by five CNN layers (CNN 1 ~ CNN 5), each layer consists the same internal structure as shown in the upper-right corner of the flow chart. The input and the output dimensions of the extracted feature maps are listed as “in” and “out” for each CNN layer. After going through the five layers and flatten, the array is merged with the BGL of the preceded round${BG}_{i-1,ref}$ (the red boxes), normalized with batch normalization, and goes through two fully connected layers (Dense) before the output, in which the number of neurons in each layer is presented in the right. For the non-pairing method (IL), the array of the preceded round in the input (the green box) and ${BG}_{i-1,ref}$(the red boxes) are not presented.

**
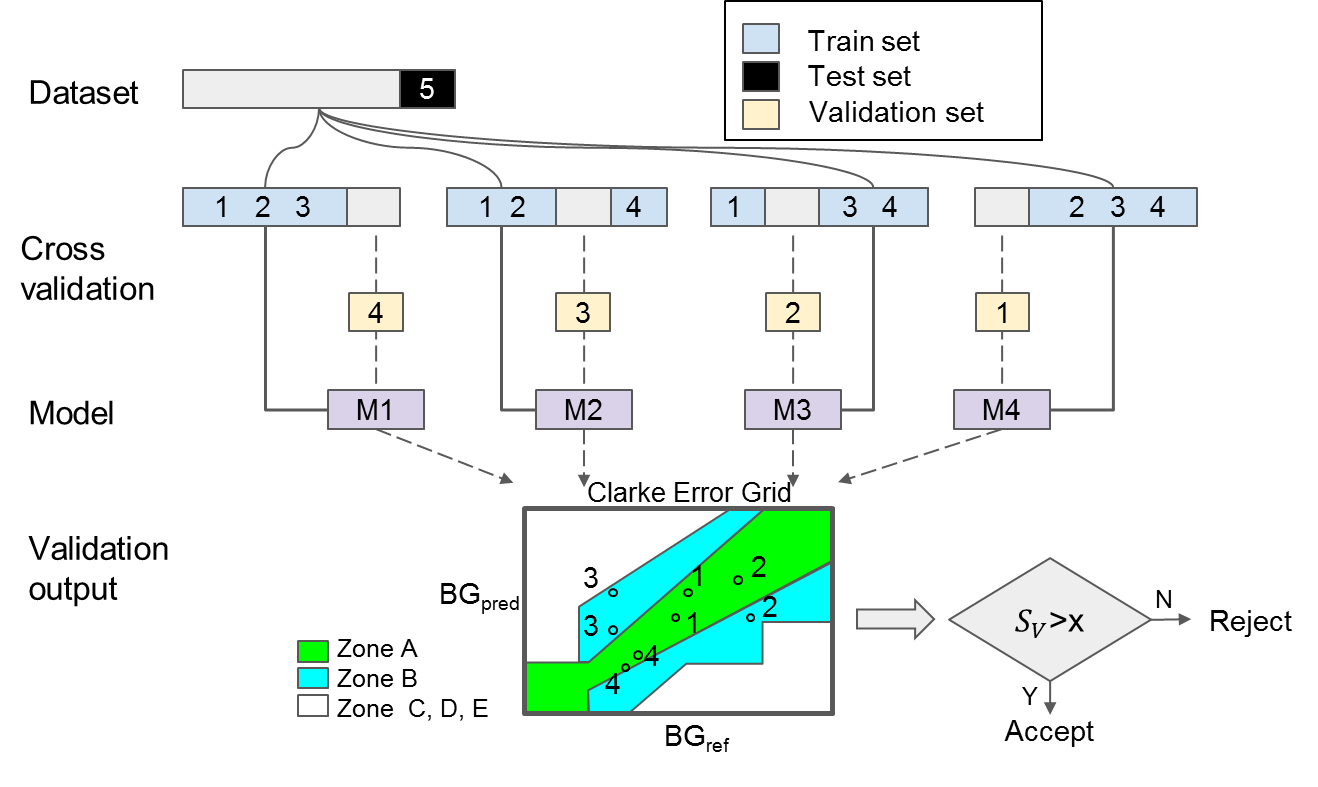
**

**Supplementary Data Figure 6| Illustration of the training and validation processes with the first stage of screening.** This example shows data of rounds 1 to 4 used for model training and validation, and data of round 5^th^ as the testing set (see descriptions in Supplementary Data Figure 7). The “leave-one-out” method successively leaves one of the rounds out of the training set during model building. As a result, four models M1~M4 are trained and then validated by their corresponding left-out round of data. By examining all the validation results in Clarke Error Grid (CEG), one obtains a validation confidence score $S_{V}$, which is used for the first stage of screening for the quality of the resulting model. The threshold value *x* is determined empirically. The numbers shown in CEG represent the model numbers of M1~M4. The repeated numeric legends are due to replicated measurements from the experiment in each round.


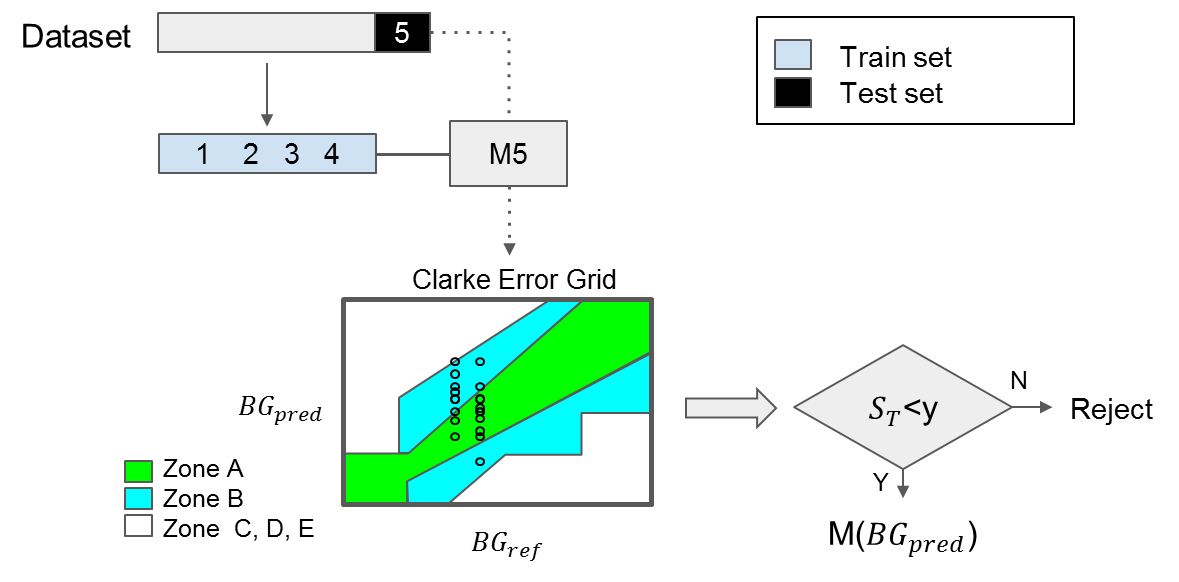


**Supplementary Data Figure 7 | Illustration of the testing processes with the second stage of screening.** When the first stage of screening is passed, the final model M5 was repeatedly trained $N$ times with the training data of all the preceded rounds from 1 ~ 4 with different random number seeds. Then the data of round 5 is tested by M5 to gather all the predictions for calculating the test spread score $S_{T}$. If $S_{T}$ passes the threshold y (systematically determined by ROC curve, see Supplementary Data Figure 8), then the median value M$({BG}_{pred})$ of all the M5 predictions is used as the final prediction.


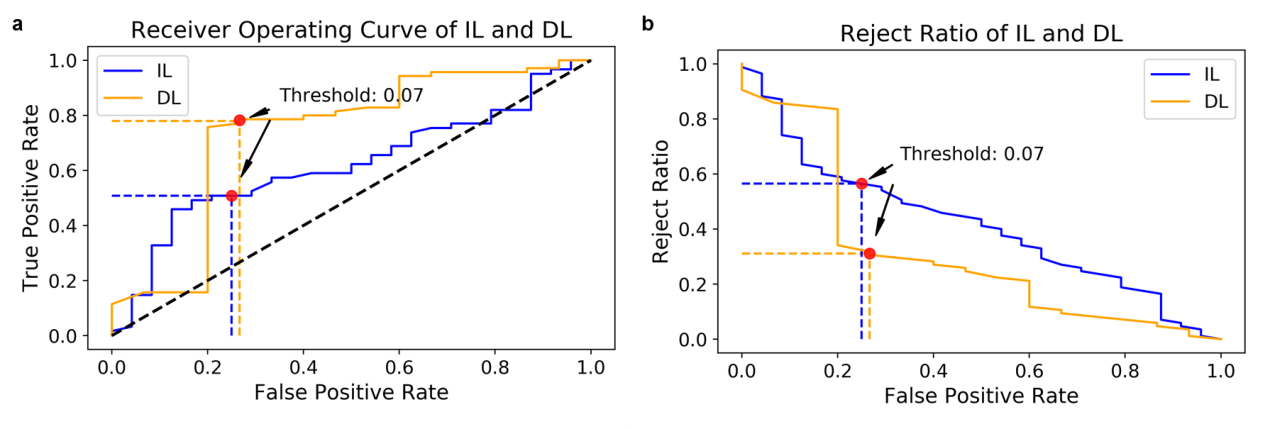


**Supplementary Data Figure 8 |** Screening threshold and pass / reject performance relationship of **(a)** ROC curve, and **(b)** reject ratio, of IL and DL models. Model training with rounds 8~15 for all the test subjects were lump-summed here. The red dots indicate how the models perform when the threshold value of screening is set at 0.07.

**
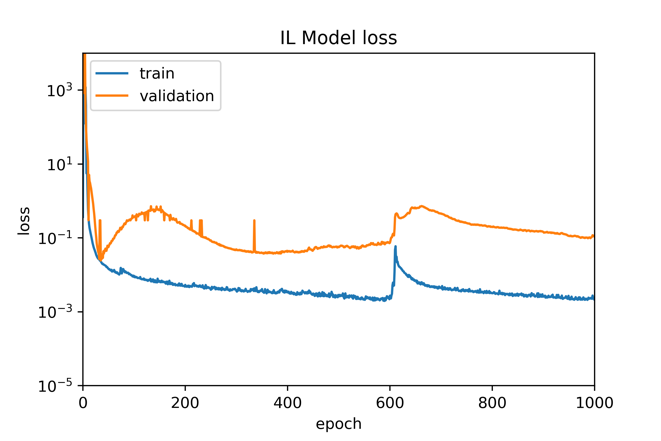

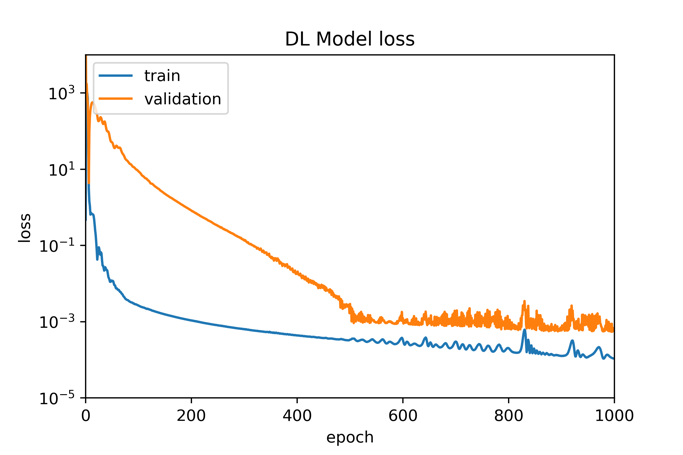
**

**Supplementary Data Figure 9 | The learning curves of IL and DL models, in log-scale.**

**
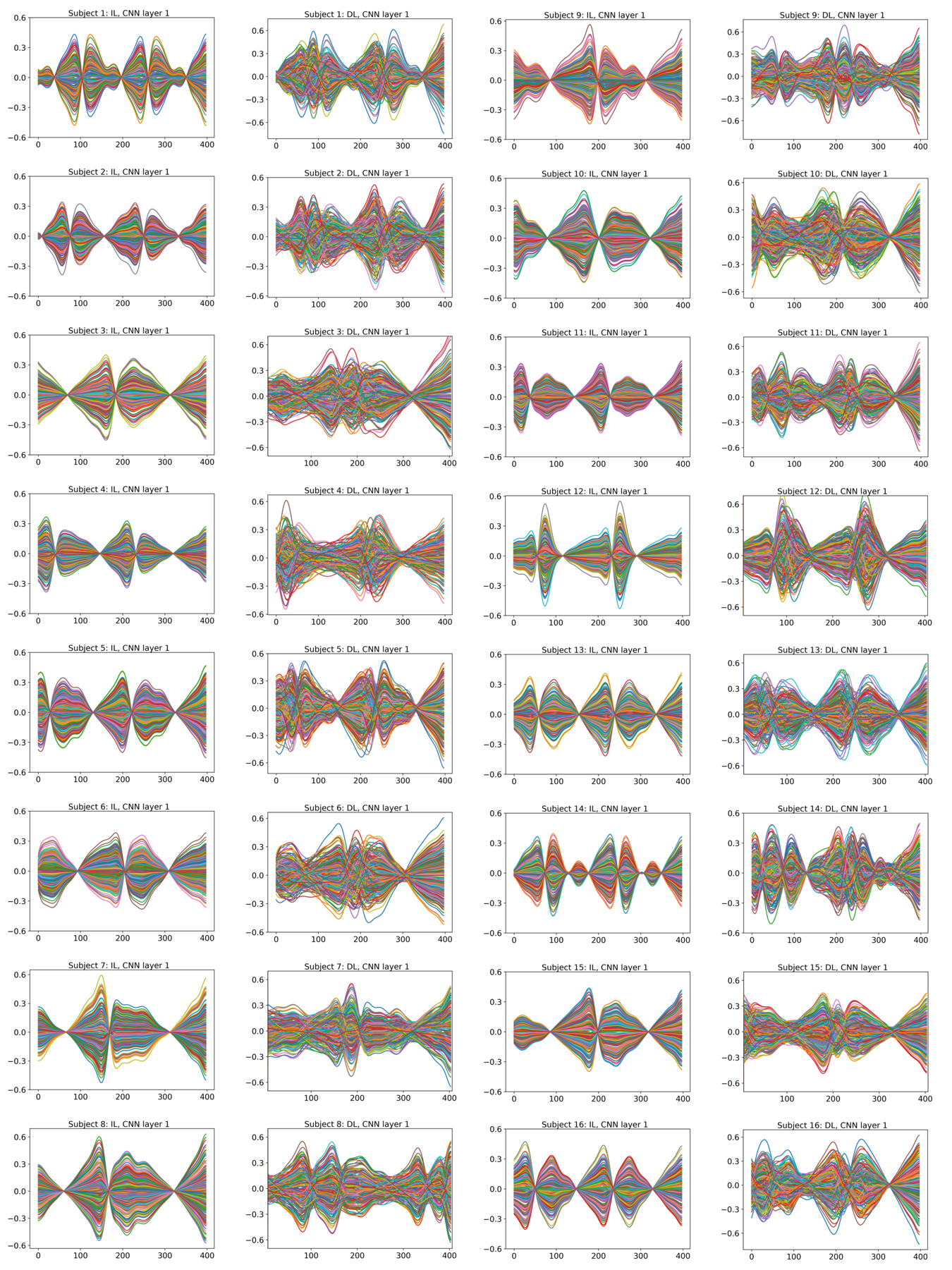
**

**Supplementary Data Figure 10 | Features captured by 256 filters of the first layer of CNN of IL and DL models for all of our recruited subjects.**

**
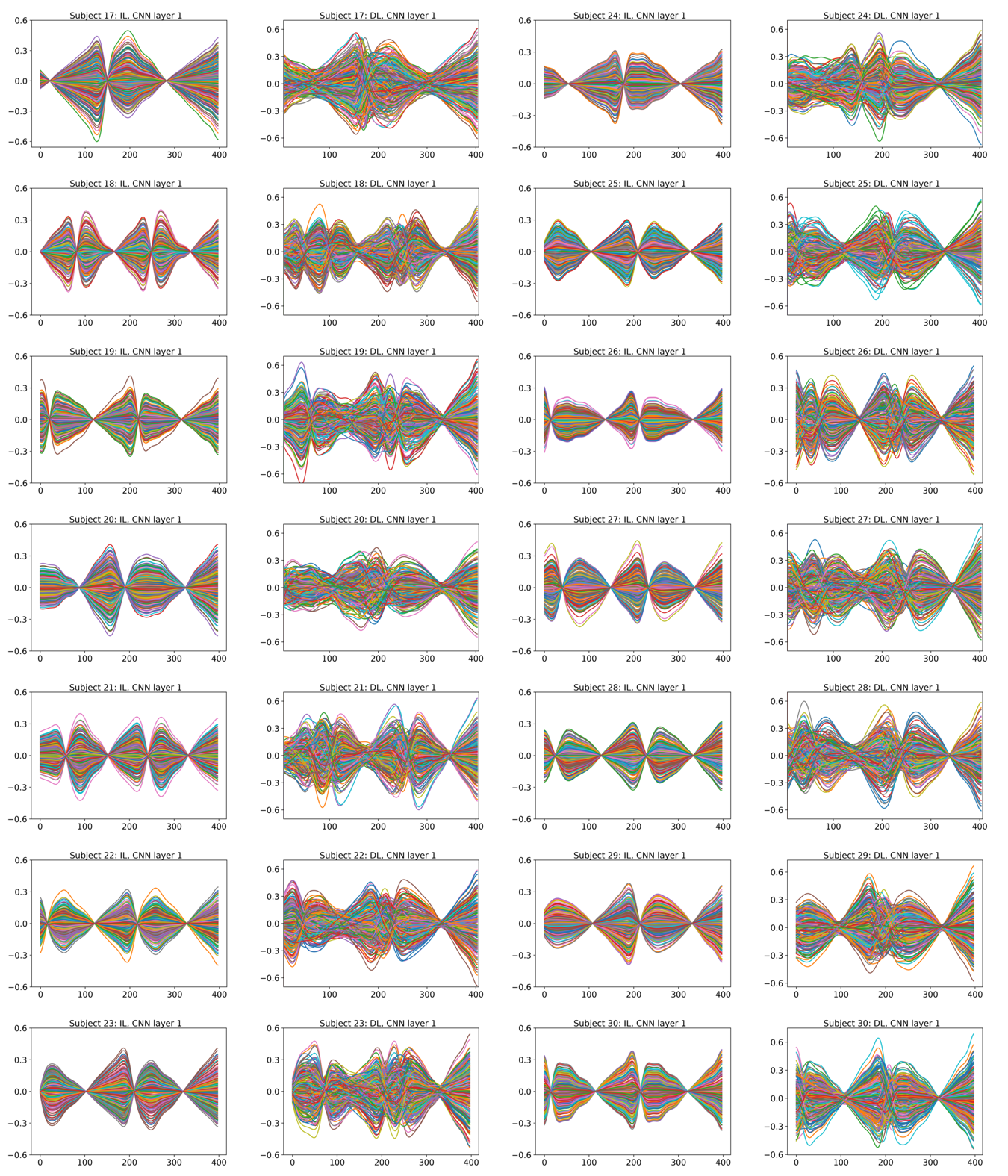
**

**Supplementary Data Figure 10 (continued) | Features captured by 256 filters of the first layer of CNN of IL and DL models for all of our recruited subjects.**

**
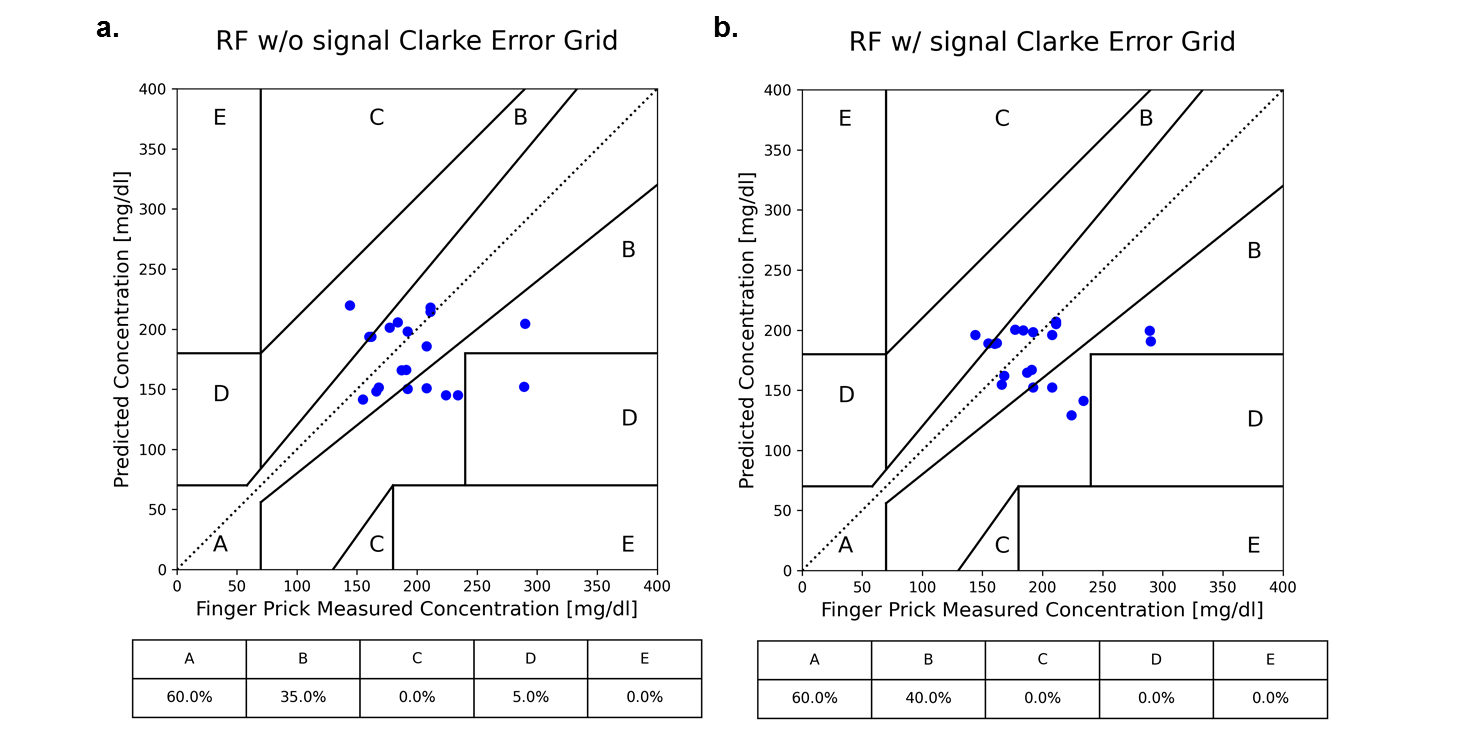
**

**Supplementary Data Figure 11 | CEG plots of BGL predictions for rounds 13~15 by the personalized Random Forest model, trained with (a) 6 morphological features data only of rounds 1~12, and (b) both 6 morphological features and PPG signals data of rounds 1~12.**

**Supplementary Data Table 1: Basic information of recruited subjects.** The last column lists number of rounds the PPG / BGL data collected from the subjects.

| **Subject No** | **Gender** | **DM Treatment** | | **Smoke** | | **Age** | **Height** | | **BMI** | **W-cir*** | **Weight** | **Repeats** |
| --- | --- | --- | --- | --- | --- | --- | --- | --- | --- | --- | --- | --- |
|  |  | **Insulin*** | **Drug** |  |  |  |  |  |  |  |  |  |
| **1** | female | 4/day | Yes | | No | 42 | | 164 | 21.5 | 77.0 | 57.8 | 15 |
| **2** | male | 1/day | Yes | | No | 53 | | 161 | 22.5 | 79.0 | 58.2 | 13 |
| **3** | female | 1/day | Yes | | No | 56 | | 156 | 20.0 | 75.0 | 48.7 | 13 |
| **4** | male | 2/day | Yes | | No | 67 | | 155 | 32.6 | 100.5 | 78.2 | 9 |
| **5** | male | 1/day | Yes | | Yes | 51 | | 174 | 33.2 | 105.0 | 100.6 | 8 |
| **6** | female | 1/day | Yes | | No | 67 | | 152 | 27.7 | 100.0 | 63.6 | 8 |
| **7** | male | 1/day | Yes | | No | 51 | | 177 | 21.8 | 78.0 | 68.4 | 7 |
| **8** | female | 4/day | Yes | | No | 66 | | 159 | 20.0 | 71.5 | 50.3 | 7 |
| **9** | male | No | No | | No | 47 | | 174 | 29.9 | 97.0 | 90.5 | 15 |
| **10** | male | No | No | | Yes | 61 | | 171 | 31.7 | 102.0 | 92.7 | 14 |
| **11** | female | No | Yes | | No | 67 | | 154 | 28.0 | 93.0 | 66.4 | 11 |
| **12** | male | No | Yes | | No | 61 | | 170 | 21.5 | 82.0 | 61.9 | 12 |
| **13** | female | No | Yes | | No | 46 | | 152 | 24.8 | 86.0 | 57.4 | 12 |
| **14** | male | No | Yes | | No | 38 | | 183 | 17.4 | 79.0 | 58.1 | 12 |
| **15** | female | No | Yes | | No | 67 | | 147 | 29.2 | 104.0 | 63.0 | 11 |
| **16** | male | No | Yes | | No | 65 | | 155 | 22.1 | 80.0 | 52.8 | 11 |
| **17** | male | No | Yes | | No | 61 | | 167 | 26.3 | 89.0 | 73.4 | 10 |
| **18** | male | No | Yes | | Yes | 62 | | 167 | 22.0 | 88.0 | 61.0 | 10 |
| **19** | female | No | Yes | | No | 58 | | 148 | 22.1 | 86.0 | 48.3 | 10 |
| **20** | male | No | Yes | | No | 72 | | 169 | 28.7 | 100.0 | 81.4 | 10 |
| **21** | male | No | Yes | | No | 72 | | 164 | 22.4 | 82.0 | 60.3 | 8 |
| **22** | female | No | Yes | | No | 60 | | 142 | 23.2 | 77.0 | 46.4 | 9 |
| **23** | male | No | Yes | | No | 67 | | 161 | 23.6 | 89.0 | 61.2 | 9 |
| **24** | male | No | Yes | | Yes | 65 | | 164 | 29.0 | 98.0 | 77.4 | 8 |
| **25** | male | No | Yes | | Yes | 60 | | 155 | 29.4 | 100.0 | 70.2 | 8 |
| **26** | female | No | Yes | | Yes | 59 | | 154 | 22.9 | 87.0 | 54.4 | 7 |
| **27** | female | No | No | | No | 64 | | 160 | 23.5 | 80.0 | 60.2 | 6 |
| **28** | female | No | Yes | | No | 76 | | 148 | 32.9 | 101.0 | 72.0 | 7 |
| **29** | female | No | Yes | | No | 63 | | 153 | 16.8 | 61.0 | 39.1 | 7 |
| **30** | male | No | Yes | | Yes | 67 | | 167 | 27.9 | 102.0 | 77.7 | 6 |

#### *Insulin: insulin injection frequency

#### *W-cir: waist circumference

**Supplementary Data Table 2. Distribution profile of 30 subjects from round 6 to round 15.**

| Round $\boldsymbol{i}^{\boldsymbol{th}}$ | # of subjects with  only $\boldsymbol{i}$rounds | # of subjects with  $\geq\boldsymbol{i}$ rounds |
| --- | --- | --- |
| 6 | 2 | 30 |
| 7 | 5 | 28 |
| 8 | 5 | 23 |
| 9 | 3 | 18 |
| 10 | 4 | 15 |
| 11 | 3 | 11 |
| 12 | 3 | 8 |
| 13 | 2 | 5 |
| 14 | 1 | 3 |
| 15 | 2 | 2 |

**Supplementary Data Table 3. Performance of 30 subjects from round 4 to round 15.** The A-Zone ratio is the ratio of data points located in the zone A of CEG plot.

| Methods | Accuracy score (mean) | Mean absolute error[mg/dl] | Root mean squared error[mg/dl] | Correlation coefficient | A-Zone ratio |
| --- | --- | --- | --- | --- | --- |
| **DL+S**  Rounds 4~15 | 84.70 | 24.07 | 32.48 | 0.725 | 77.7% |
| **DL**  Rounds 4~15 | 80.62 | 30.98 | 42.94 | 0.640 | 65.5% |
| **IL**  Rounds 4~15 | 76.29 | 37.42 | 50.93 | 0.554 | 56.7% |
| **DL+S**  Rounds 4~7 | 81.90 | 28.57 | 37.45 | 0.646 | 69.4% |
| **DL+S**  Rounds 8~11 | 86.84 | 20.38 | 27.09 | 0.812 | 85.1% |
| **DL+S**  Rounds 12~15 | 93.87 | 10.35 | 12.17 | 0.960 | 100% |
| **DL**  Rounds 4~7 | 77.68 | 34.04 | 43.78 | 0.606 | 56.8% |
| **DL**  Rounds 8~11 | 83.90 | 28.09 | 44.27 | 0.674 | 76.9% |
| **DL**  Rounds 12~15 | 87.70 | 21.69 | 30.44 | 0.782 | 80.6% |
| **IL**  Rounds 4~7 | 76.11 | 36.10 | 46.44 | 0.578 | 55.1% |
| **IL**  Rounds 8~11 | 76.54 | 39.28 | 57.83 | 0.540 | 58.2% |
| **IL**  Rounds 12~15 | 76.50 | 39.16 | 51.58 | 0.417 | 61.1% |

**Supplementary Data Table 4. Training time and resource usage of each model.**

| Training rounds | IL (sec) | DL (sec) | DL+S (sec) | # of GPUs usage | GPU RAM usage (GB) |
| --- | --- | --- | --- | --- | --- |
| 4 | 3519 | 3525 | 4322 | 1 | < 11 |
| 5 | 3526 | 3529 | 4734 | 1 | < 11 |
| 6 | 3558 | 3562 | 5181 | 1 | < 11 |
| 7 | 3582 | 3594 | 5673 | 2 | 11 ~ 22 |
| 8 | 3659 | 3680 | 6160 | 2 | 11 ~ 22 |
| 9 | 3701 | 3723 | 6667 | 3 | 22 ~ 33 |
| 10 | 3746 | 3775 | 7179 | 3 | 22 ~ 33 |

**Supplementary Data Code 1. Pseudo code of Induction Learning.**

# Input: i_train: The model will be trained by rounds 1 to i_train.

# i_test: The model will be tested by i_test, i_test < i_train.

# PPG_raw[:]: PPG raw signal of each round.

# Output: prediction: The predicted BG of round i_train.

def IL_model(i_train, i_test, PPG_raw):

# Data preprocessing.

for i in (1 to all_rounds):

# Use Butterworth filter and Bigger-Fall-Side algorithm to get the high

# frequency part of PPG_raw_signal, and annotate the valleys and peaks

# of the PPG waveform (Supplementary Data Figure 3)

PPG, Valley_idx, Peak_idx <- PPG_Raw_Signal_Analy(PPG_raw[i])

# Extract windows (1.6s backwardly from each valley) and features. Each

# window j of round i contains the following data (Supplementary Data Figure 4)

# Signal_vec[j,i] consists: PPG signal of the window

# Feature_vec[j,i] consists: HR_rate,Area,FW_25,FW_50,FW_75,FW_100

# Window[j,i] <- (Signal_vec[j,i], Feature_vec[j,i])

Window[:,i] <- Window_Create(PPG, Vally_idx, Peak_idx)

# Collect the data for training and testing.

i_train_set <- (1 to i_train)

test_data <- Window[:,i_test]

train_data <- Window[:,i_train_set]

# Train the model by IL learning (Figure 1a, Supplementary Data Figure 5)

# Input: train_data: training data.

# test_data: testing data, to do prediction.

# Output: BG_pred: after training, output the prediction of test_data

BG_pred = model_IL_train(train_data, test_data)

return prediction <- BG_pred

**Supplementary Data Code 2. Pseudo code of Deduction Learning.**

# Input: i_train: The model will be trained by rounds 1 to i_train.

# i_test: The model will be tested by i_test, i_test < i_train.

# PPG_raw[:]: PPG raw signal of each round.

# BG_ref[:]: Measured BG (ground truth) of rounds 1 to i_train.

# Output: prediction: The predicted BG of round i_train.

def DL_model(i_train, i_test, PPG_raw, BG_ref):

# Data preprocessing.

for i in (1 to all_rounds):

# Use Butterworth filter and Bigger-Fall-Side algorithm to get the high

# frequency part of PPG_raw_signal, and annotate the valleys and peaks

# of the PPG waveform (Supplementary Data Figure 3)

PPG, Valley_idx, Peak_idx <- PPG_Raw_Signal_Analy(PPG_raw[i])

# Extract windows (1.6s backwardly from each valley) and features. Each

# window j of round i contains the following data (Supplementary Data Figure 4)

# Signal_vec[j,i] consists: PPG signal of the window

# Feature_vec[j,i] consists: HR_rate,Area,FW_25,FW_50,FW_75,FW_100

# Window[j,i] <- (Signal_vec[j,i], Feature_vec[j,i])

Window[:,i] <- Window_Create(PPG, Vally_idx, Peak_idx)

# Create window pairs from round i and round i-1 (Supplementary Data Figure 4)

for i in (2 to all_rounds):

for j in (index of all windows in round i):

jj <- randomly_selected(1 to j)

Window_pair[j, 1,i-1] <- Window[j,i]

Window_pair[jj,2,i-1] <- Window[jj,i-1]

# Collect the paired data for training and testing.

i_train_set <- (1 to i_train-1)

test_pair <- Window_pair[:,:,i_test]

train_pair <- Window_pair[:,:,i_train_set]

# Train the model by DL learning (Figure 1b, Supplementary Data Figure 5)

# Input: train_pair: training data.

# test_pair: testing data, to do prediction.

# BG_ref: ground truth, as input to DL model for training.

# Output: BG_pred: after training, output the prediction of test_pair.

BG_pred = model_DL_train(train_pair, test_pair, BG_ref[i_train_set])

return prediction <- BG_pred

**Supplementary Data Code 3. Pseudo code of Deduction Learning with screening.**

# Input: i_train: The model will be trained by rounds 1 to i_train.

# i_test: The model will be tested by i_test, i_test < i_train.

# PPG_raw[:]: PPG raw signal of each round.

# BG_ref[:]: Measured BG (ground truth) of rounds 1 to i_train.

# Output: prediction: The predicted BG of round i_train.

def DL_screening(i_train, i_test, PPG_raw, BG_ref):

# Data preprocessing.

for i in (1 to all_rounds):

# Use Butterworth filter and Bigger-Fall-Side algorithm to get the high

# frequency part of PPG_raw_signal, and annotate the valleys and peaks

# of the PPG waveform (Supplementary Data Figure 3)

PPG, Valley_idx, Peak_idx <- PPG_Raw_Signal_Analy(PPG_raw[i])

# Extract windows (1.6s backwardly from each valley) and features. Each

# window j of round i contains the following data (Supplementary Data Figure 4)

# Signal_vec[j,i] consists: PPG signal of the window

# Feature_vec[j,i] consists: HR_rate,Area,FW_25,FW_50,FW_75,FW_100

# Window[j,i] <- (Signal_vec[j,i], Feature_vec[j,i])

Window[:,i] <- Window_Create(PPG, Vally_idx, Peak_idx)

# Create window pairs from round i and round i-1 (Supplementary Data Figure 4)

for i in (2 to all_rounds):

for j in (index of all windows in round i):

jj <- randomly_selected(1 to j)

Window_pair[j, 1,i-1] <- Window[j,i]

Window_pair[jj,2,i-1] <- Window[jj,i-1]

# Collect the paired data for training and testing.

test_pair <- Window_pair[:,:,i_test]

train_pair <- Window_pair[:,:,(1 ... i_train-1)]

# Model training: cross validation (Supplementary Data Figure 6)

for i in (1 to i_train-1):

# Separate the training data into training set and validating set.

i_train_set <- (1 to i_train-1 except i)

vali_train <- train_pair[:,i_train_set]

vali_data <- train_pair[:,i]

# Train the model by DL learning (Figure 1b, Supplementary Data Figure 5)

# Input: vali_train: training data.

# vali_data: validation data

# BG_ref: ground truth, as input to DL model for training.

# Output: BG_pred: after training, output the prediction of vali_data

BG_pred = model_DL_train(vali_train, vali_data, BG_ref[i_train_set])

# Collect the prediction and the corresponding ground truth.

vali_pred.append(BG_pred)

vali_ref.append(BG_ref[i+1])

# Confidence score S_V (Supplementary Data Figure 6) is computed by Eq.2, i.e.,

# the number of predictions in zone A and zone B of CEG plot with weightings

# wA=1, wB=0.5, and then normalized by the total number of predictions. The

# more predictions in zone A or zone B, the higher the confidence score.

confidence_score <- Confidence_Score(vali_ref, vali_pred)

# The 1st stage of screening, where confidence_score_thrd is emperically

# set to 50 and 60 for round <= 5 and round > 5, respectively.

if (confidence_score < confidence_score_thrd):

reject

else:

# Model testing: the final model is repeatedly trained 10 times with various

# random numbers , in order to get spread_score S_T (Supplementary Data Figure 7)

for i in (1 to 10):

BG_pred <- model_DL_train(train_pair, test_pair, BG_ref[(1 to i_train-1)])

p.append(BG_pred)

# p is the collection of predictions repeated 10 times of testing data.

p.remove(max(p), min(p))

# The 2nd stage of screening, where spread_score_thrd is determined by

# ROC curve analysis (Supplementary Data Figure 8)

spread_score <- Standard_deviation(p)/median(p)

if (spread_score > spread_score_thrd):

reject

else:

return prediction <- median(p)
